# Cell uptake and intracellular trafficking of bioreducible poly(amidoamine) nanoparticles for efficient mRNA translation in chondrocytes

**DOI:** 10.3389/fbioe.2023.1290871

**Published:** 2023-11-10

**Authors:** Adriano P. Pontes, Steffen van der Wal, Saketh R. Ranamalla, Karin Roelofs, Ioan Tomuta, Laura B. Creemers, Jaap Rip

**Affiliations:** ^1^ 20Med Therapeutics BV, Leiden, Netherlands; ^2^ Department of Pharmaceutical Technology and Biopharmacy, University of Medicine and Pharmacy “Iuliu Hațieganu”, Cluj-Napoca, Romania; ^3^ Department of Orthopedics, University Medical Center Utrecht, Utrecht, Netherlands

**Keywords:** non-viral gene delivery, nanoparticle, mRNA delivery, poly(amidoamine), osteoarthritis, chondrocyte

## Abstract

Disulfide-containing poly(amidoamine) (PAA) is a cationic and bioreducible polymer, with potential use as a nanocarrier for mRNA delivery in the treatment of several diseases including osteoarthritis (OA). Successful transfection of joint cells with PAA-based nanoparticles (NPs) was shown previously, but cell uptake, endosomal escape and nanoparticle biodegradation were not studied in detail. In this study, C28/I2 human chondrocytes were transfected with NPs co-formulated with a PEG-polymer coating and loaded with EGFP mRNA for confocal imaging of intracellular trafficking and evaluation of transfection efficiency. Compared with uncoated NPs, PEG-coated NPs showed smaller particle size, neutral surface charge, higher colloidal stability and superior transfection efficiency. Furthermore, endosomal entrapment of these PEG-coated NPs decreased over time and mRNA release could be visualized both *in vitro* and in live cells. Importantly, cell treatment with modulators of the intracellular reducing environment showed that glutathione (GSH) concentrations affect translation of the mRNA payload. Finally, we applied a D-optimal experimental design to test different polymer-to-RNA loading ratios and dosages, thus obtaining an optimal formulation with up to ≈80% of GFP-positive cells and without toxic effects. Together, the biocompatibility and high transfection efficiency of this system may be a promising tool for intra-articular delivery of therapeutical mRNA in OA treatment.

## 1 Introduction

Osteoarthritis (OA) is a musculoskeletal disorder affecting the synovial joint that leads to severe pain and progressive loss of joint function, representing a huge socioeconomic burden (Cui et al., 2020). The OA progression is significantly related to inflammation and oxidative stress (Henrotin et al., 2005). Current pharmacological therapies aim to relieve pain and control inflammation (Zhang et al., 2016). However, their efficacy is limited and they have a high incidence of long-term adverse effects (Mukherjee et al., 2011). The development of nucleic acid-based therapies–such as messenger RNA (mRNA) or small interfering RNA (siRNA)–has shown great potential to modulate pathophysiological pathways at the level of protein synthesis, across different joint tissues that drive OA progression (Wijesinghe et al., 2021). One of the challenges for systemic or local delivery of nucleic acids is their potential degradation by exonucleases in the extracellular space. For this reason, entrapment of nucleic acids in delivery systems provides protection and improves circulation time (Colella et al., 2020). Nanoparticles (NPs) are promising candidates for intra-articular delivery of nucleic acids. Their small size is an advantage for penetration in the extracellular matrix of the cartilage, which consists of an intricate network of collagen type II fibers and proteoglycans with a pore size around 60 nm (Rothenfluh et al., 2008). However, despite recent advances, there is a lack of clinical trials investigating nanoparticle-based treatments for joint diseases such as OA (Anselmo and Mitragotri, 2021).

In this context, cationic nanoparticles based on synthetic polymers, such as poly(amidoamine)s (PAMAM dendrimers or linear PAAs), have emerged as promising carriers for gene therapies. This is a result of their bioresponsive and biodegradable properties, good water solubility, reproducible manufacturing process, and structural versatility for both loading and further chemical functionalization (Arote et al., 2011; Sun et al., 2017). Recently, linear PAA-based NPs loaded with tdTomato mRNA showed successful, dose-dependent transfection of bovine chondrocytes (bCH) in 2D cultures and 3D pellet cultures (Sturm et al., 2021). However, the nanoparticle fate after crossing the plasma membrane remained partly unexplored what makes it difficult to identify potential bottlenecks and thus to optimize these carriers further for higher efficacy.

The cationic charge of the PAA polymer, provided by the ionizable amine groups in the polymer backbone, allows for efficient condensation of nucleic acids by electrostatic interactions in aqueous solution (Lin et al., 2007). The resulting cationic nanoparticles interact with the negatively charged cell membrane resulting in an efficient cell uptake. Although cationic NPs are easily taken up by cells *in vitro* due to their relatively high positive surface charge, the *in vivo* application may be hampered by limited biodistribution and possible side effects after administration (Knudsen et al., 2015; Sukhanova et al., 2018). A commonly used approach to minimize these negative effects and to improve the *in vivo* applicability is the shielding of the NP surface using polyethylene glycol (PEG) (D’souza and Shegokar, 2016). To this end, we introduced an anionic block copolymer composed of poly-L-glutamic acid and PEG (PGA_7.5k_-PEG_5k_) in the PAA-based NP formulation. After endocytosis, nanoparticles need to escape the endosomes, what has been identified as one of the major bottlenecks in gene delivery (Pei and Buyanova, 2019). Although still under debate, the “proton sponge effect” remains the most generally accepted mechanism for endosomal escape of cationic polymers (Freeman et al., 2013). This hypothesis is associated with the large buffering capacity of polycations such as PAA, which become protonated during endosome maturation resulting in Cl^−^ and H_2_O influx. This results in endosome swelling and rupture, releasing the payload into the cytoplasm. The particular PAA system described in this paper contains quinoline (Q) moieties to further increase the transfection efficiency. The parent molecule chloroquine, an anti-malarial drug, is a known additive in *in vitro* transfections, increasing its efficiency through several mechanisms including interaction with the nucleotide payload and endosomal membranes (Hajimolaali et al., 2021). These linear PAAQ polymers are covalently assembled around a core of multi-armed ethylenediamine (Mw 800, 2% w/w) to form a pre-organized polymeric scaffold (ps-PAAQ), as a precursor for the formation of the mRNA-loaded nanoparticles. The redox-sensitive disulfide bridges in the polymer allow for release of nucleic acids from the NPs under the reducing conditions found in the cytosol, due to the presence of relatively high concentrations of glutathione (GSH).

In this study we evaluated nucleic acid delivery of PEG-coated ps-PAAQ nanoparticles in C28/I2 human chondrocytes, using EGFP mRNA as a payload. The studies were performed to obtain further insight into the uptake and the intracellular behavior of these NPs. The stages of the intracellular trafficking were studied using fluorescently labeled ps-PAAQ NPs. Firstly, we evaluated internalization of uncoated *versus* PEG-coated NPs as the coating may impact electrostatic interactions with the negatively charged cell membrane, and thus affect uptake as well as intracellular trafficking. Secondly, endosomal leakage/escape over time were monitored and quantified by confocal microscopy using fluorescent probes (calcein and LysoTracker™). Thirdly, the payload release was evaluated *in vitro* and in live cells over time to visualize the separation of mRNA and polymers. To the best of our knowledge, this is the first imaging of the intracellular bioreduction of disulfide-containing PAA-based NPs together with visualization of reporter expression. In addition, we evaluated modulators of the intracellular reducing environment to determine the effect of glutathione depletion in GFP translation. Finally, we maximized the transfection efficiency in the C28/I2 chondrocytes by optimizing the polymer-to-RNA loading ratio and dosage using a systematic Design of Experiments (DoE) approach.

## 2 Materials and methods

### 2.1 Synthesis of the ps-PAAQ polymers

Two monomers were made for the synthesis of the ps-PAAQ polymers. The first monomer, cystamine bis(acrylamide) (CBA), was synthesized as described by Lin *et al.* (Lin et al., 2006) The second monomer, N1-(7-chloroquinolin-4-yl)-hexane-1,6-diamine (Q6), was synthesized analogously to the described synthesis by Natarajan et al. (2008) Other chemicals were purchased and used without further purification from Sigma-Aldrich or Avantor.

The ps-PAAQ (or p (CBA-ABOL-Q)/PEI) were synthesized by Michael-type polymerization of primary amines with bis-acrylamides as described by Lin *et al.* (Lin et al., 2006) In brief, a small round-bottom flask was charged with 4-aminobutanol **(ABOL)** (0.60 g, 6.73 mmol), bis(acrylamide) **(CBA)** (2.60 g, 10 mmol), and N1-(7-chloroquinolin-4-yl)-hexane-1,6-diamine **(Q6)** (0.60 g, 2.16 mmol). MeOH (10 mL) was added, followed by a solution of CaCl_2_ (0.44 g, 4 mmol) in water (2 mL). The resulting suspension was heated to 50°C and was allowed to stir for an additional 48 h resulting in a nearly translucent solution (PAAQ polymer). A solution of branched PEI800 (Sigma-Aldrich Mn 600, 100 mg/mL in water, 680 μL) was then added and the reaction mixture was allowed to stir for another 72 h with heating. The reaction was subsequently terminated by acidification to pH 4 using hydrochloric acid and the resulting solution was transferred directly into a dialysis membrane (Spectrapor 6, 10 kD MWCO) for purification, where the polymer was dialyzed extensively against water. Then, the material was filtered through a 0.45 µm filter (cellulose acetate) and ultimately lyophilized to yield the branched ps-PAAQ polymer as a (hygroscopic) white solid.

^1^H NMR (400 MHz, DMSO-d6) 1.25–1.75 (multiple peaks), 2.4–3.05 (multiple peaks), 3.08–3.45 (multiple peaks) 5.53–5.65 (m, -C=CHH^1^), 6.01–6.16 (m, -C=CH^1^H), 6.77–6.88 (bs), 7.64–7.76 (bs), 8.00–8.15 (bs), 8.33–8.61 (large bs), 8.66–8.81 (bs), 9.45–9.68 (bs) ppm.

The ratio of integrals at the 1–2 ppm range to the integral at 6.8 ppm were determined to be 20:1 corresponding to a feedstock of approximately 1–3 parts Q:ABOL.

The ps-PAAQ without disulfide bonds in the polymer backbone (or p (HMBA-ABOL-Q)/PEI) was synthesized as described above, except by replacing the CBA by hexamethylenediamine bisacrylamide (HMBA) (2.24 g, 10 mmol).

### 2.2 Labeling of azide-functionalized ps-PAAQ with sulfo-Cy5 DBCO

The synthesis of the PAAQ polymer was performed as above but in addition, after the 72 h of reaction time with PEI800, 11-azido-3,6,9-trioaxaundecan-1-amine (0.33 g, 1.5 mmol) was added directly to the reaction mixture and was subsequently allowed to stir for another 24 h to functionalize remaining acrylamide moieties with an azide functionality. The reaction mixture was then acidified and purified as described above. A small multiplet peak appears on ^1^H-NMR at 3.72 ppm while the acrylamide signal is concomitantly decreased compared with non-functionalized polymer.

Fifty milligrams of azide-functionalized ps-PAAQ was dissolved in a mixture of water/MeOH (1.1 mL, 10:1 v/v) and sulfo-Cy5 DBCO (Lumiprobe GmbH, 50 µL of a 20 mg/mL solution in DMSO) was added and allowed to stir 1.5 h. The reaction mixture was then transferred to dialysis tubing (Spectrapor 6, 10 kD MWCO) and extensively dialyzed against water. After filtration through a 0.45 µm filter (cellulose acetate) followed by freeze drying, a blue solid (20 mg) was obtained that contained no free Cy5 when assayed by TLC (30% MeOH in CH_2_Cl_2,_ R_f_ = 0.3). 20 mg of the polymer corresponded to 0.6 mg sulfo-Cy5 when analyzed with absorbance spectroscopy (absorbance at 650 nm, *ε* = 2.5 · 10^5^ mol^-1^ L^-1^) (Mujumdar et al., 1993).

### 2.3 Nanoparticle formulation and physicochemical characterization

The ps-PAAQ polymers were mixed with EGFP mRNA (Cleanup, TriLink Biotechnologies) in 10 mM Histidine 10% Trehalose buffer (pH 6.5) to obtain nanoparticles. The mRNA concentration was kept constant in all formulations (60 μg/mL), with increasing concentrations of ps-PAAQ polymers (0.75 mg/mL - 3 mg/mL) resulting in a target ps-PAAQ:mRNA loading ratio ranging from 12.5 to 50 w/w (respective N/P ratio of 10:1 to 40:1). For the formulation of Cy5-labeled nanoparticles, a ratio of 1:9 w/w Cy5-labeled ps-PAAQ to non-labeled ps-PAAQ was used.

To obtain PGA-PEG-coated nanoparticles, the coating material (mPEG_5k_-b-PLE_50_, Alamanda Polymers) was added to the mRNA solution in the first step, which was then added to the polymers in the same mixing step, using a 1:1 coating to ps-PAAQ w/w ratio.

The resulting nanoparticle size, zeta potential and particle concentration were measured using Multi-Angle Dynamic Light Scattering (MADLS) in the Zetasizer Ultra (Malvern), with three different angles: 175°, 90° and 13°. Samples were diluted ten-fold in 10 mM Histidine 10% Trehalose buffer (pH 6.5) and loaded in a low-volume quartz cuvette (ZEN2112, Malvern). Results were analyzed in the ZS Explorer software (version 3.0, Malvern). The measurements for Cy5-labeled nanoparticles and the experiment comparing the size of nanoparticles in formulation buffer *versus* culture medium were performed in the Zetasizer Nano ZS90 (Malvern), with a 90-degree scattering optics. Results were analyzed in the Zetasizer software (version 7.13, Malvern).

### 2.4 Cell culture

Immortalized C28/I2 human chondrocytes were cultured in a growth medium composed of Dulbecco’s modified Eagle’s medium (DMEM GlutaMAX™, high glucose, pyruvate; Gibco), supplemented with 10% v/v fetal bovine serum (FBS; Biowest), 100 units/mL penicillin and 100 μg/mL streptomycin (P/S; Gibco) at 37°C under a humidified 5% CO_2_ atmosphere. Medium changes were performed every 3 days and cells were passaged at 70%–90% confluency at a seeding density of 6,000 cells/cm^2^ in 175 cm^2^ T-flasks.

### 2.5 Transfection of C28/I2 cells

For the transient transfection experiments, C28/I2 cells were seeded at 20,000 cells/cm^2^ in 48 well-cell culture plates (Sarstedt) and allowed to attach overnight, using growth medium.

The ps-PAAQ NPs nanoparticles loaded with EGFP mRNA were initially suspended in serum-free DMEM without antibiotics, supplemented with 20 mM sterile HEPES buffer (Gibco). After gently mixing, the NP suspensions were dropwise added to the cells to a total volume of 0.2 mL/well. Following incubation at 37°C for 4 h, the serum-free medium was replaced by the full growth medium containing 10% v/v FBS and antibiotics. Finally, 24 h after transfection, fluorescent images were taken, and the percentage of transfected cells was calculated as described in the next section. For confocal imaging, cells were cultured in 35 mm CELLview™ glass-bottom dishes (Greiner Bio-One) and seeded at 20,000 cells/cm^2^ under the same culture conditions with a total volume of 0.2 mL/well. Lipofectamine™ MessengerMAX™ Transfection Reagent (Invitrogen) was used as a positive control for mRNA transfection, following the manufacturer’s protocol. Live cell imaging was performed in an environmentally controlled chamber (37°C and 5% CO_2_) in all experiments.

### 2.6 Image acquisition and processing

Transfected efficiency was evaluated by fluorescence microscopy after 24 h of incubation (THUNDER Imager; Leica). Before imaging, the nuclei from all cells were stained with 5 µM Hoechst 33342 (Invitrogen) for 5 min at 37°C, followed by three washing steps with DMEM. Subsequently, staining with 0.25 µM SYTOX™ Orange Nucleic Acid Stain (Invitrogen) was carried out for 20 min at 37°C, as a marker of dead cells (Truernit and Haseloff, 2008). A total of three fields were imaged per well and each experiment was done in triplicate (3 wells/condition) from 3 independent cultures. Between 8,000 and 12,000 cells were quantified per condition and independent culture. The number of positive cells per channel was calculated by automatic cell counting on ImageJ (version 1.53; National Institutes of Health) as previously reported (Grishagin, 2015).

Software analysis was carried out by “batch processing” according to Macro functions from the Supplementary Material, in order to automatically count the number of positive cells per channel. Transfection efficiency was expressed as the percentage of GFP-positive cells per Hoechst-positive cells. Cell viability was calculated as the percentage of SYTOX-negative cells per Hoechst-positive cells, compared to untreated cells.

### 2.7 Cell uptake

For verifying the effect of PEGylation on NP internalization efficiency, PEG-coated and uncoated Cy5-labeled ps-PAAQ NPs were incubated with C28/I2 cells in a 35 mm glass-bottom dish (CELLview™), during 3 h at 37°C. For this, we used a loading ratio of 25 w/w ps-PAAQ:mRNA and dosage of 480 ng mRNA per well. To visualize cell boundaries, the plasma membrane was stained with 2.5 μg/mL CellMask™ Orange (Invitrogen) for 5 min at 37°C, followed by three washing steps with DMEM. Confocal images were acquired immediately after washing, using a confocal laser microscope (SPX8; Leica) and 63x/1.4 oil-immersion objective. The Cy5-labeled NPs were visualized with 649 nm excitation wavelength, while CellMask-stained plasma membrane was imaged by using a 554 nm laser. The Z-stack method, changing the focal length from the bottom to the top of a single cell, was used to provide an orthogonal view of the cell thickness as an evidence of particle internalization. Image processing was conducted using ImageJ to estimate the number of internalized nanoparticles within the cell boundaries. After background subtraction, a single-cell area was manually selected using the selection tool to estimate the mean fluorescence intensity per μm^2^.

In the uptake kinetics study, we used FACS to quantify the percentage of cells with internalized nanoparticles over time. In summary, cells were trypsinized with 100 μL of Trypsin-EDTA (0.25%) per well and incubated for 3 min at 37°C. Then, 400 μL of DMEM with 10% v/v FBS was added to each well and the cells were resuspended. Cells from three technical replicates were pooled into a single Eppendorf tube and centrifuged for 5 min at 300xg. The medium was aspirated, and the pellet was resuspended in 2% paraformaldehyde (PFA) for fixation, during 10 min at room temperature. The cells were again centrifuged for 5 min at 300xg, and the pellets were resuspended in 100 µL fresh DPBS/0.5% BSA FACS buffer. The samples were transferred to a 96-well plate and kept on ice until measurement was performed in a MACSQuant^®^ flow cytometer (Miltenyi Biotec). Forward, side scatter, and laser voltage were adjusted using untreated cells. Recording conditions were set to collect 10,000 live, single cell events per sample.

### 2.8 Intracellular trafficking: endosomal escape

For evaluating endosomal leakiness, C28/I2 cells were incubated for 15 min at 37°C with PEG-coated ps-PAAQ NPs or 100 µM chloroquine (Sigma-Aldrich). After this period, cells were washed once with DMEM to remove the residual extracellular particles and then stained with calcein (Invitrogen) at a self-quenching concentration of 3 mM for 15 min, as previously reported (Vermeulen et al., 2018). The cells were washed three times with DMEM and incubated for a period of 3 h at 37°C before imaging. Confocal images were acquired using a confocal laser microscope (SPX8; Leica) and 63x/1.4 oil-immersion objective.

In order to track the intracellular fate of the nanoparticles over time, PEG-coated ps-PAAQ NPs were co-loaded with EGFP mRNA and silencer Cy3-labeled negative control siRNA (Invitrogen) at a 9:1 w/w ratio. These fluorescently labeled NPs were incubated with C28/I2 cells seeded on 35 mm glass-bottom dishes (CELLview™) for imaging at timepoints 3, 24 and 48 h. For this, we used a loading ratio of 25 w/w ps-PAAQ:mRNA and dosage of 480 ng mRNA per well. Three hours after incubation, the dishes were washed once with DMEM to remove the residual extracellular particles. Fresh growth medium supplemented with 10% v/v FBS and antibiotics was added to cells for optimal growth, then the dishes were placed back in the incubator for measurements after 24 and 48 h. For the imaging of acidic compartments, endo/lysosomes were stained with 50 nM Lysotracker™ Deep Red (Invitrogen) in DMEM for 15 min, followed by three washing steps with DMEM. LysoTracker™ staining was performed before imaging at each timepoint. Confocal images were acquired immediately after washing, using a confocal laser microscope (SPX8; Leica) and 63x/1.4 oil-immersion objective. The LysoTracker-stained endo/lysosomes were visualized with 649 nm excitation wavelength, while Cy3 siRNA-loaded NPs were imaged by using a 554 nm laser.

Images were processed using ImageJ and the frequency of endosomal escape events over time was calculated by the “Colocalization Finder” plugin in ImageJ. After single cell area selection, the Pearson correlation coefficient (PCC) values were estimated from the colocalization of the fluorescence intensities from both channels (Dunn et al., 2011). PCC readouts ranged from 0 to 1. Values close to zero indicate that the fluorescence intensities of the two channels are uncorrelated, whereas values close to 1 indicate that the two fluorescence intensities are directly and linearly related. Threshold PCC values, related images and descriptions are reported in Supplementary Figure S1.

### 2.9 Nanoparticle disassembly and mRNA release in cells

The *in vitro* release of mRNA from NPs was verified by agarose gel electrophoresis, using 2% agarose gel and SYBR^®^ Safe DNA Gel Stain (Invitrogen) for visualization of the EGFP mRNA. In this assay, the following samples were loaded in the gel: free mRNA, 10 µL of NPs loaded with mRNA, and 10 µL loaded NPs treated for 5 min at 60°C with both 2 M 1,4-Dithiothreitol (DTT; Sigma-Aldrich) and 5 mg/mL heparin (Sigma-Aldrich). Gel electrophoresis was performed for 30 min at 100 V and visualized using a ChemiDoc Imaging System (Bio-Rad). If mRNA is not released from nanoparticles following treatment, it does not migrate in the gel.

For simultaneous monitoring of nanoparticle disassembly and cargo release over time, Cy5-labeled PEG-coated NPs were co-loaded with EGFP mRNA and AZDye568-EGFP mRNA (RiboPro) at a 9:1 w/w ratio. These nanoparticles were incubated with C28/I2 cells in a 35 mm glass-bottom dish (CELLview™) for imaging at timepoints 3, 8 and 24 h. For this, we used a loading ratio of 25 w/w ps-PAAQ:mRNA and dosage of 480 ng mRNA per well. Three hours after incubation, the dishes were washed once with DMEM to remove the residual extracellular particles. Fresh growth medium supplemented with 10% v/v FBS and antibiotics was added to cells for optimal growth, then the dishes were directly imaged or placed back in the incubator for measurements after 8 and 24 h (without additional washing steps). Images were acquired using a confocal laser microscope (SPX8; Leica) and 63x/1.4 oil-immersion objective. The Cy5-labeled NPs were visualized with 649 nm excitation wavelength, while the AZDye568-EGFP mRNA was imaged by using a 577 nm laser. The GFP protein fluorescence was detected with a 488 nm laser line.

### 2.10 Effect of modulators of the intracellular reducing environment

The C28/I2 cells were treated with specific chemical enhancers and inhibitors to investigate the effects on transfection efficiency of nanoparticles. To determine the effect of modulating the intracellular reducing environment on GFP expression, cells were incubated either in the presence of 20–2000 µM DL-buthionine sulfoximine (BSO; Sigma-Aldrich) for 24 h prior to transfection, or 1–2 mM glutathione–monoethyl ester (GSH–MEE; Sigma-Aldrich) for 3 h after medium change, 4 h after transfection. Cell culture and transfection were carried out in DMEM without pyruvate (high glucose, Gibco), as previously recommended in studies involving oxidative stress (Babich et al., 2009).

### 2.11 Validation of optimal formulation

The optimal formulation of ps-PAAQ NPs for transfection in C28/I2 cells was found by the Design of Experiments (DoE) method (Eriksson et al., 2008). A D-optimal design was chosen, due to the precise estimation of factor effects and the small number of experimental trials compared to standard factorial design. The key variables that could influence transfection efficiency were defined as the polymer-to-mRNA ratio (in w/w) and the dosage of mRNA per well (in ng). The DoE was developed by using the MODDE software (version 13, Sartorius Stedim Data Analytics AB), comprising of 10 runs and 3 center points, therefore 13 experiments.

### 2.12 Statistics

Statistical analyses were performed using GraphPad Prism (version 9.0; GraphPad^®^ Software). For comparing differences between two groups, parametric data were evaluated through unpaired *t*-test, while non-parametric data were evaluated through Mann-Whitney test. For comparing differences among more than two groups, we used one-way analysis of variance (ANOVA) followed by the Tukey’s *post hoc* multiple comparisons test. Alternatively, Dunnett’s test was used when comparing the mean of experimental groups with a single control group, and Bonferroni’s test was applied when there was a set of planned comparisons beforehand. Values of *p* ≤ 0.05 were considered to be statistically significant. All data are shown as mean ± standard deviation.

## 3 Results

### 3.1 Nanoparticle size and zeta potential

The ps-PAAQ polymers were co-formulated with EGFP mRNA and PGA_7.5k_-PEG_5k_ to obtain nanoparticles (designated “PEG-coated NPs”), at several loading ratios ranging from 12.5 to 50 w/w (ps-PAAQ:mRNA, N/P ratio 10:1 to 40:1). For the intracellular studies in the following sections, we used the 25 w/w loading ratio (N/P ratio 20:1) based on previous results with different cell lines (unpublished data). The amount of loaded mRNA was kept constant at 60 μg/mL for all formulations. MADLS measurements confirmed the formation of nanoparticles, with an average size around 60 nm and a monodisperse distribution for PEG-coated NPs (Table 1 and Supplementary Figure S2). However, a larger particle size and multiple resolvable peaks were observed for uncoated NPs (Table 1 and Supplementary Figure S2). It was also possible to observe a proportional increase in particle concentration with increasing polymer concentrations in the coated formulation (from 12.5 to 50 w/w ratio) (Table 1). Zeta potential showed near-neutral values for coated NPs, as an effect of the hydrophilic PGA_7.5k_-PEG_5k_ addition, while uncoated NPs had a strongly positive zeta potential of +32.7 mV. The characterization of Cy5-labeled NPs used in this study is shown in Supplementary Table S1.

**TABLE 1:** Particle characterization by Multi-Angle Dynamic Light Scattering (MADLS). Results are the combined data of three individual measurements. All nanoparticles were loaded with 60 μg/mL of EGFP mRNA. See Supplementary Figure S2 for particle size distributions.

NP type	ps-PAAQ:mRNA ratio (w/w)	Peak 1 mean by intensity (nm)	Polydispersity index (PDI)	Zeta potential (mV)	Concentration (x10^12^ particles/mL)
Coated	12.5	63.8	0.260 ± 0.033	−1.4 ± 0.4	1.04
Coated	25	60.3	0.162 ± 0.010	−2.5 ± 0.3	2.54
Coated + Cy3-siRNA	25	60.1	0.156 ± 0.020	−1.0 ± 0.1	3.39
Coated	42.5	56.7	0.149 ± 0.038	+0.8 ± 0.2	5.66
Coated	50	59.3	0.146 ± 0.014	−2.4 ± 0.1	5.78
Uncoated	25	137.7	0.233 ± 0.071	+32.7 ± 2.5	N/A^a^

^a^ Low data quality assessment–multiple resolvable size populations in the sample (Supplementary Figure S2).

**FIGURE 1:**
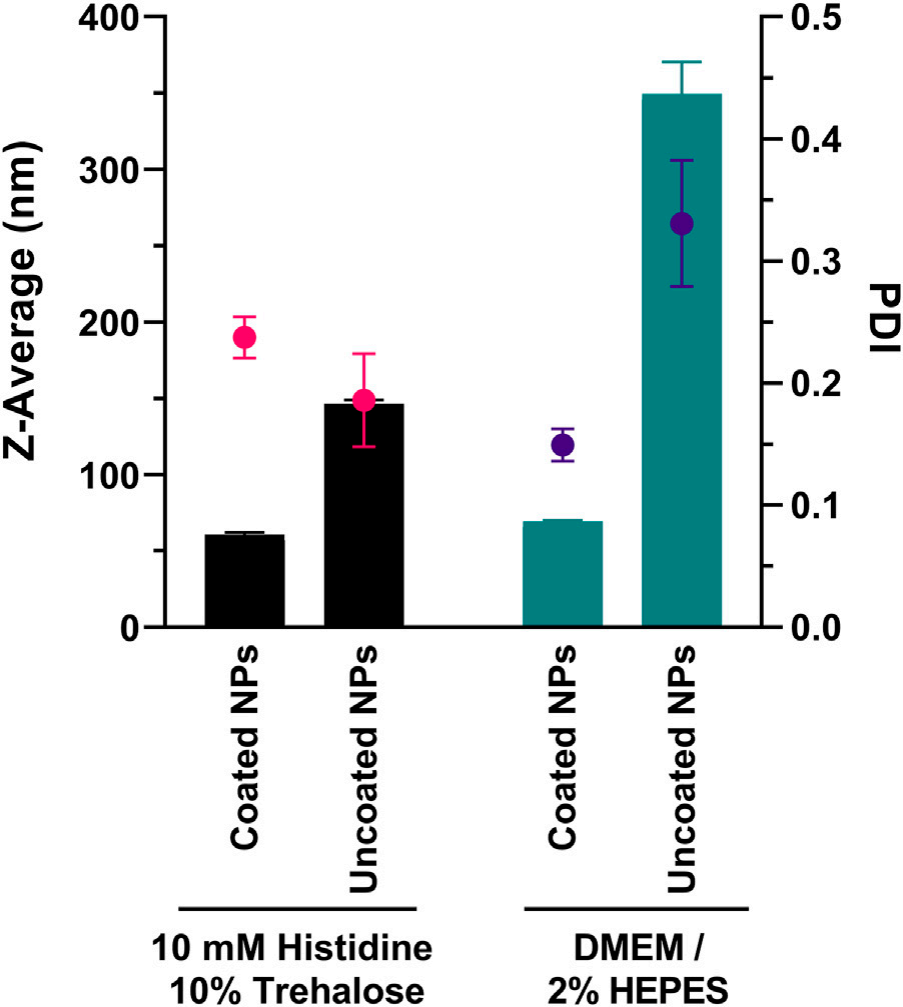
Particle size after incubation of ps-PAAQ NPs (25 w/w loading ratio) with formulation buffer or culture medium. DMEM was supplemented with 2% HEPES buffer and is serum-free. Bars represent Z-Average (in nm) and spots represent polydispersity index (PDI). Results are the combined data of three individual measurements.

In the following, we assessed the mean diameter size of PEG-coated and uncoated NPs in presence of culture medium (serum-free) used in transfections. The formulations (25 w/w loading ratio) were analyzed by DLS after mixing with 10 mM Histidine 10% Trehalose (formulation buffer) or DMEM/2% HEPES medium. In both conditions, a 1:1 v/v dilution ratio was used. Figure 1 shows the results following incubation for 30 min at room temperature.

The incubation in 10 mM Histidine 10% Trehalose buffer did not impact the original diameter of nanoparticles, compared to the measurements from Table 1: PEG-coated NPs showed an average size of 60.6 ± 1.5 nm (PDI 0.237 ± 0.017), and uncoated ps-PAAQ NPs an average size of 146.5 ± 2.4 nm (PDI 0.186 ± 0.038). While incubation with DMEM did not substantially affect the size of coated NPs (69.4 ± 0.7 nm; PDI 0.149 ± 0.013), it did cause a substantial increase in particle size and polydispersity for uncoated NPs (349.5 ± 20.9 nm; PDI 0.331 ± 0.052). This corresponds to over a two-fold increase in size compared with uncoated NPs in 10 mM Histidine 10% Trehalose buffer. The respective particle size distributions are shown in Supplementary Figure S3. Besides, the cryogenic electron microscopy (Cryo-EM) of the PEG-coated NPs in buffer was obtained for morphology and size confirmation (Supplementary Figure S4).

### 3.2 Cell uptake and transfection

**FIGURE 2:**
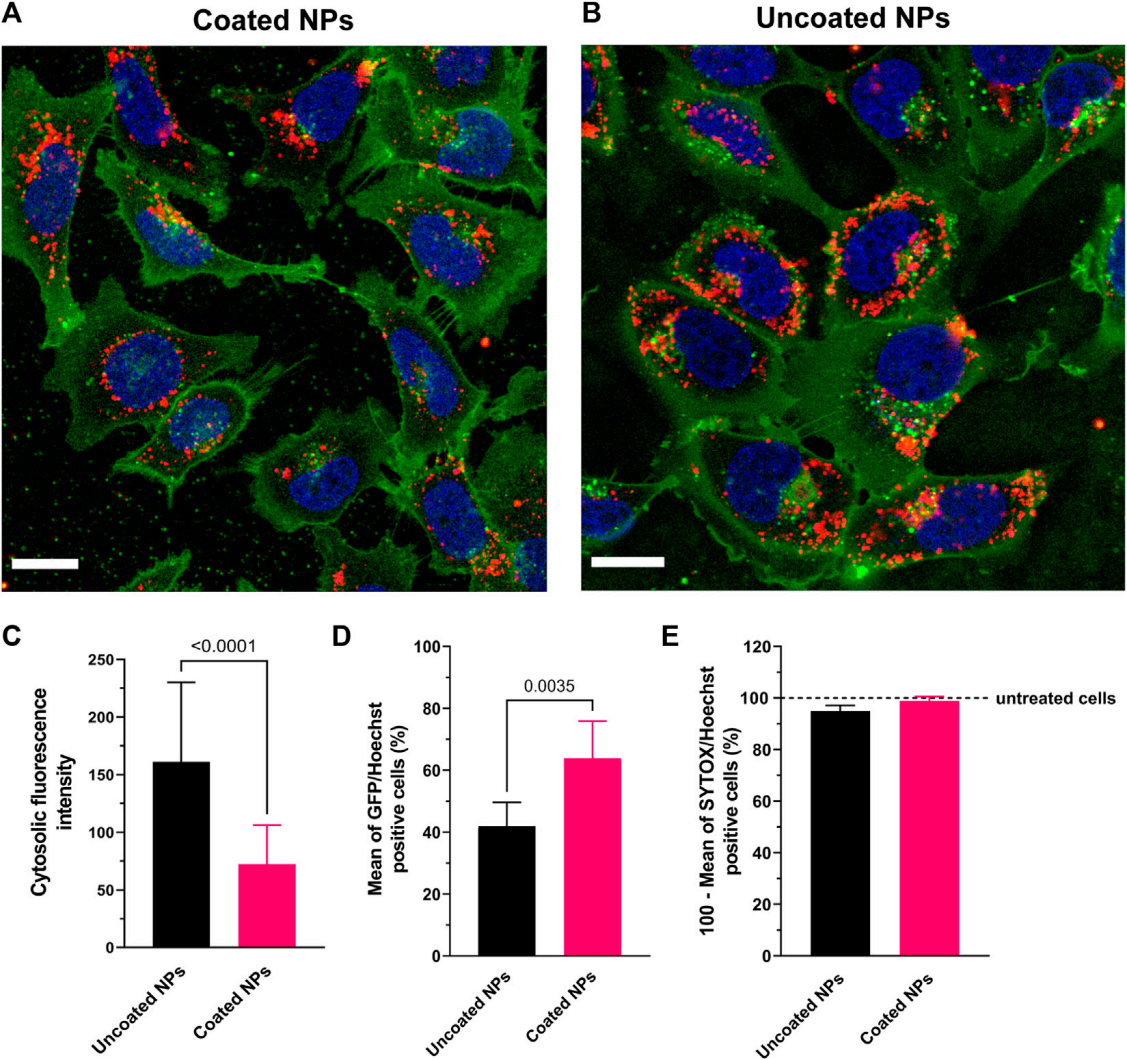
Uptake of ps-PAAQ NPs in C28/I2 human chondrocytes. **(A, B)** Uptake in C28/I2 cells after 3 h of incubation with Cy5-labeled coated or uncoated NPs (in red). The plasma membrane is stained with CellMask™ Orange and digitally pseudo-colored in green for a higher contrast against the NPs in red. Scale bar: 20 μm. **(C)** Comparison of uptake efficiency between coated *versus* uncoated NPs, as measured by the integrated density of Cy5 signal per cell on ImageJ software (13–15 cells per group, *n* = 3, Mann-Whitney test, *p*-value ≤0.05). **(D)** Transfection efficiency as expressed by the percentage of GFP-positive cells (8,000–12,000 cells per group, *n* = 3, unpaired *t*-test, *p*-value ≤0.05). **(E)** Viability of C28/I2 chondrocytes after 24 h of transfection using uncoated or PEG-coated NPs, compared to untreated cells (8,000 to 12,000 cells per group, *n* = 3). SYTOX™ Orange Nucleic Acid Stain was combined with Hoechst 33342 for two-color observation of live and dead cells.

To evaluate the internalization efficiency of nanoparticles, we followed the cellular uptake of neutral PEG-coated NPs into C28/I2 human chondrocytes, compared to positively charged uncoated NPs. Both nanoparticle types were labeled with water-soluble sulfo-Cy5 dye to track their subcellular localization in the cells. With confocal microscopy after 3 h of incubation, these fluorescent NPs appeared as punctate spots that agglomerate preferentially in the perinuclear region (shown in red in Figures 2A, B). The orthogonal sectioning and reconstruction of the z-stacks shows the clear proof that the particles were internalized (Supplementary Figure S5). While a large amount of both uncoated and PEG-coated NPs were detected virtually inside all cells, the punctate spots corresponding to uncoated ps-PAAQ NPs appear larger and more intense compared with PEG-coated nanoparticles (Figures 2A, B). For quantifying the uptake efficiency of nanoparticles, the cytosolic fluorescence intensity was measured using the ImageJ software. Indeed, uncoated ps-PAAQ NPs showed a two-fold increase in cellular uptake compared with PEG-coated NPs (Figure 2C). In contrast, transfection efficiency–as measured by percentage of GFP-positive cells–with coated NPs (64% ± 12%) was significantly higher than results obtained with uncoated NPs (42% ± 8%) (Figure 2D). Cell viability was ≥95% for transfections with both nanoparticle types (Figure 2E).

We also evaluated the uptake kinetics of uncoated *versus* PEG-coated ps-PAAQ NPs over time. The percentage of C28/I2 cells with internalized nanoparticles was measured by FACS per hour, in the first 4 h after transfection. The PEG-coated NPs showed a slower uptake kinetics compared with uncoated NPs, especially in earlier timepoints (Supplementary Figure S6). This finding corroborates the uptake efficiency results obtained by confocal microscopy above.

The localization studies in the next sections were performed with PEG-coated NPs (same loading ratio and payload quantity), given their improved stability and lower polydispersity in cell culture medium, as well as superior GFP protein expression in C28/I2 chondrocytes compared with uncoated ps-PAAQ NPs.

### 3.3 Intracellular trafficking: endosomal escape

**FIGURE 3:**
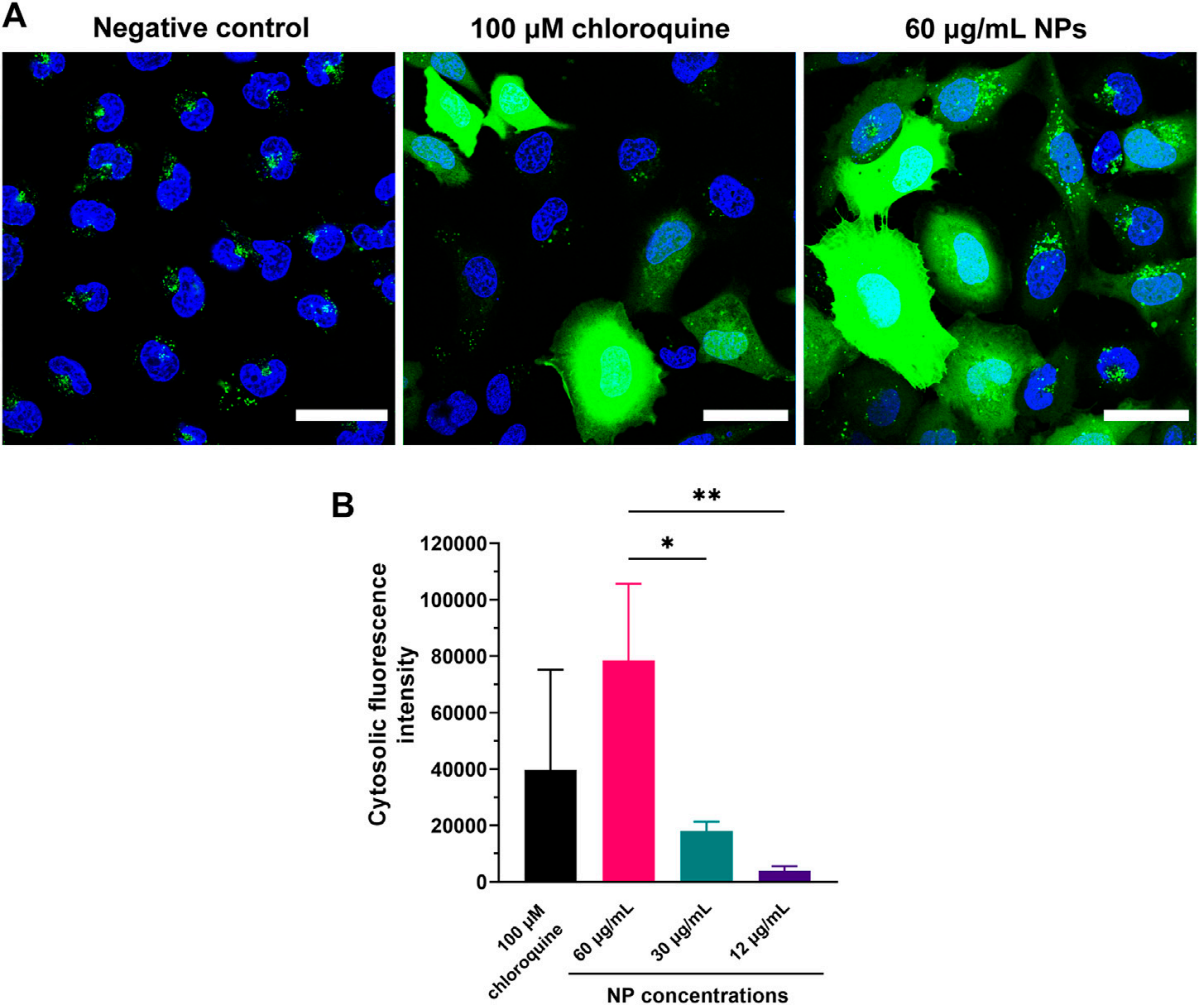
Evaluation of endosomal leakiness induced by coated ps-PAAQ NPs. **(A)** C28/I2 cells were incubated with nanoparticles and calcein at a self-quenching concentration of 3 mM for 3 h. Left picture represents the negative control (cells incubated with calcein, without prior addition of ps-PAAQ NPs). Center picture represents the positive control (cells incubated with calcein after addition of 100 µM chloroquine). Right picture shows C28/I2 cells incubated with calcein post-transfection with ps-PAAQ NPs, using a loading ratio of 25 w/w ps-PAAQ:mRNA and nanoparticle concentration of 60 μg/mL. Scale bar: 40 μm. **(B)** The cytosolic fluorescence intensity of calcein was quantified from microscopy images after incubation with 60, 30 and 12 μg/mL of ps-PAAQ NPs. Calcein fluorescence was corrected for negative control (only calcein). In total, 52–58 cells were analyzed per group (One-Way ANOVA followed by Tukey’s test). Statistically significant difference is indicated by (*) to *p*-value ≤0.05, or (**) to *p*-value ≤0.01.

PEG-coated ps-PAAQ nanocarriers were further explored regarding their ability to induce endosomal rupture. To evaluate the role of endosomal membrane leakiness on the escape of nanoparticles, calcein was incorporated into endosomes as a marker to study endosome integrity. Because it was used in a self-quenching concentration (3 mM), a subtle leak in the endosomal membrane could be easily observed by the change from a punctate fluorescent pattern (endosomes) to a diffuse (dequenched) cytoplasmic fluorescence of higher intensity. Chloroquine is a well-described endosomal escape enhancer (Hajimolaali et al., 2021), which functions as positive control for endosomal escape. A chloroquine derivative is also incorporated into the ps-PAAQ polymer, where it was expected to similarly enhance endosomal escape. Indeed, calcein release can be clearly observed in both cells that have taken up nanoparticles as well as those that were treated with chloroquine (Figure 3A). In addition, Figure 3B shows the intensity of cytosolic calcein fluorescence after incubation with 60, 30 and 12 μg/mL of ps-PAAQ NPs, clearly indicating that calcein release is a concentration-dependent effect.

**FIGURE 4:**
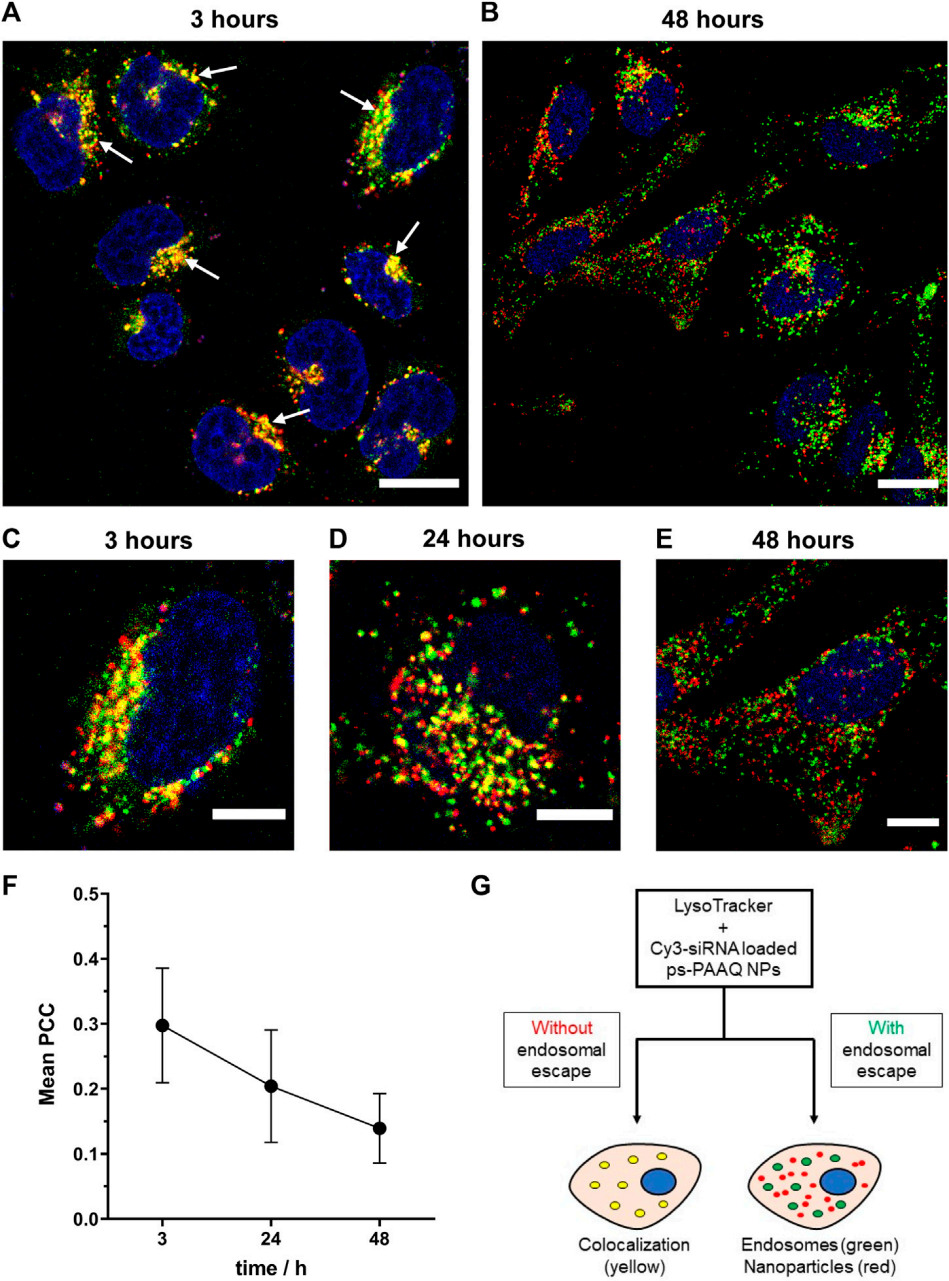
Intracellular trafficking of coated ps-PAAQ NPs over time. **(A, B)** Fluorescence images of C28/I2 cells incubated with Cy3-siRNA loaded ps-PAAQ NPs at different timepoints (3 and 48 h). Red channel represents ps-PAAQ NPs, green channel shows LysoTracker-stained endo/lysosomes, and yellow color indicated by arrows (3 h timepoint) represents colocalization. Scale bar: 20 µm. **(C–E)** Higher magnification, fluorescence images of C28/I2 cells incubated with Cy3-siRNA loaded ps-PAAQ NPs after 3, 24 and 48 h of transfection. Scale bar: 10 µm. **(F)** Colocalization coefficient between the fluorescence signal of Cy3-siRNA loaded ps-PAAQ NPs and LysoTracker™ (7–13 cells per timepoint, *n* = 3). PCC analysis was performed by using ImageJ software. **(G)** Schematic of endosomal escape visualization using the endo/lysosomal probe LysoTracker™ Deep Red.

In order to evaluate endosomal escape over time, PEG-coated NPs were co-loaded with a mixture of EGFP mRNA and Cy3-labeled siRNA, and subsequently incubated with C28/I2 cells for 3, 24 and 48 h. Before imaging at each timepoint, endo/lysosomes were stained using LysoTracker™ Deep Red (Figure 4G). After 3 h of incubation, yellow punctuate spots indicating colocalization of the two labels (Figures 4A, C) clearly show endosomal entrapment of NPs at this timepoint. The average PCC value was maximum 3 h post-transfection (0.30 ± 0.09) in this experiment (Figure 4F). The fraction of particles colocalizing with the acidic compartments decreased after 24 h (Figure 4D), when more ps-PAAQ NPs were found to be no longer entrapped inside the endo/lysosomal vesicles (PCC 0.20 ± 0.09). In the first two timepoints (3 and 24 h), a higher heterogeneity in the intracellular localization was observed between different cells, explaining the large standard deviation (SD) of the mean PCC values (Figure 4F). After 48 h, nearly all ps-PAAQ NPs were observed to be distributed in the cytosol (Figures 4B, E), suggesting an effective escape of ps-PAAQ NPs from the acidic compartments (PCC 0.14 ± 0.05). This represents a 50% decrease in the endosomal entrapment compared with the initial 3 h timepoint. These results constitute further evidence that ps-PAAQ nanoparticles induce the rupture of the endo/lysosomes and escape to the cytosol over time.

### 3.4 Nanoparticle disassembly and mRNA release

**FIGURE 5:**
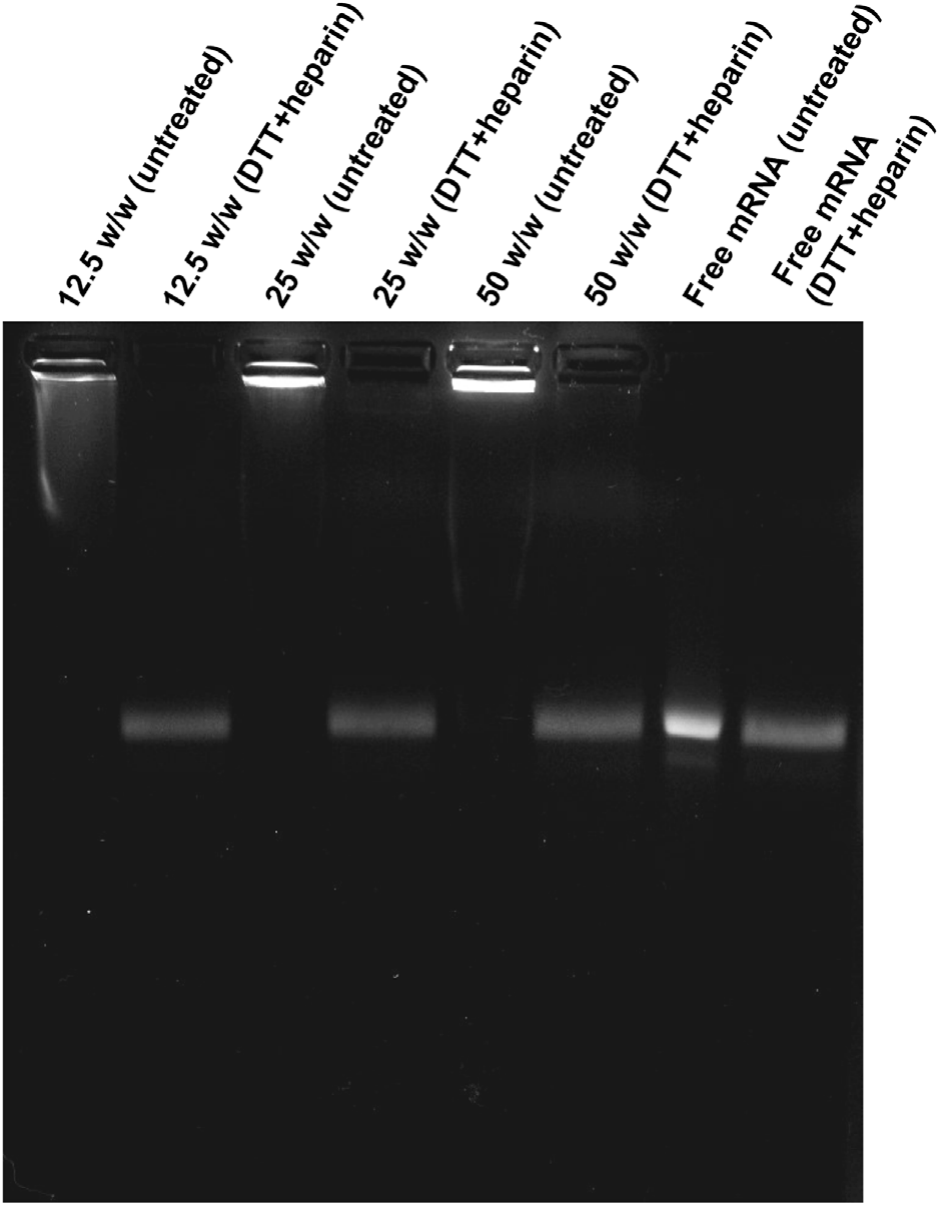
Migration of EGFP mRNA from coated ps-PAAQ NPs in agarose gel electrophoresis. Three different loading ratios (w/w ps-PAAQ:mRNA): 12.5 (lanes 1–2); 25 (lanes 3–4); and 50 (lanes 5–6). Free mRNA (60 μg/mL) was loaded on lanes 7–8 as a control. The second well of each group was treated with DTT and heparin to release mRNA by reduction and displacement, respectively.

The *in vitro* release of mRNA from PEG-coated ps-PAAQ NPs was investigated by gel electrophoresis, using heparin as a competing polyanion (Oupickí et al., 2001) in combination with the reducing agent dithiothreitol (DTT) to facilitate mRNA release through disulfide exchange-mediated ps-PAAQ fragmentation (van der Aa et al., 2011). Three different loading ratios were tested to compare the effect of increasing NP concentrations on mRNA release, ranging from 12.5 to 50 (w/w) ps-PAAQ:mRNA. After treatment of ps-PAAQ NPs with 2 M DTT and 5 mg/mL heparin before electrophoresis, the mRNA release was monitored on agarose gel. Gel electrophoresis showed strong mRNA binding for all polymer-to-mRNA ratios, since untreated nanoparticles prevented mRNA migration in the gel (Figure 5

). In contrast, when treated with 5 mg/mL heparin and 2 M DTT, a single band corresponding to free mRNA was observed for all loading ratios. Together these results indicate that the loaded mRNA was released from PEG-coated NPs in a simulated reducing environment.

**FIGURE 6:**
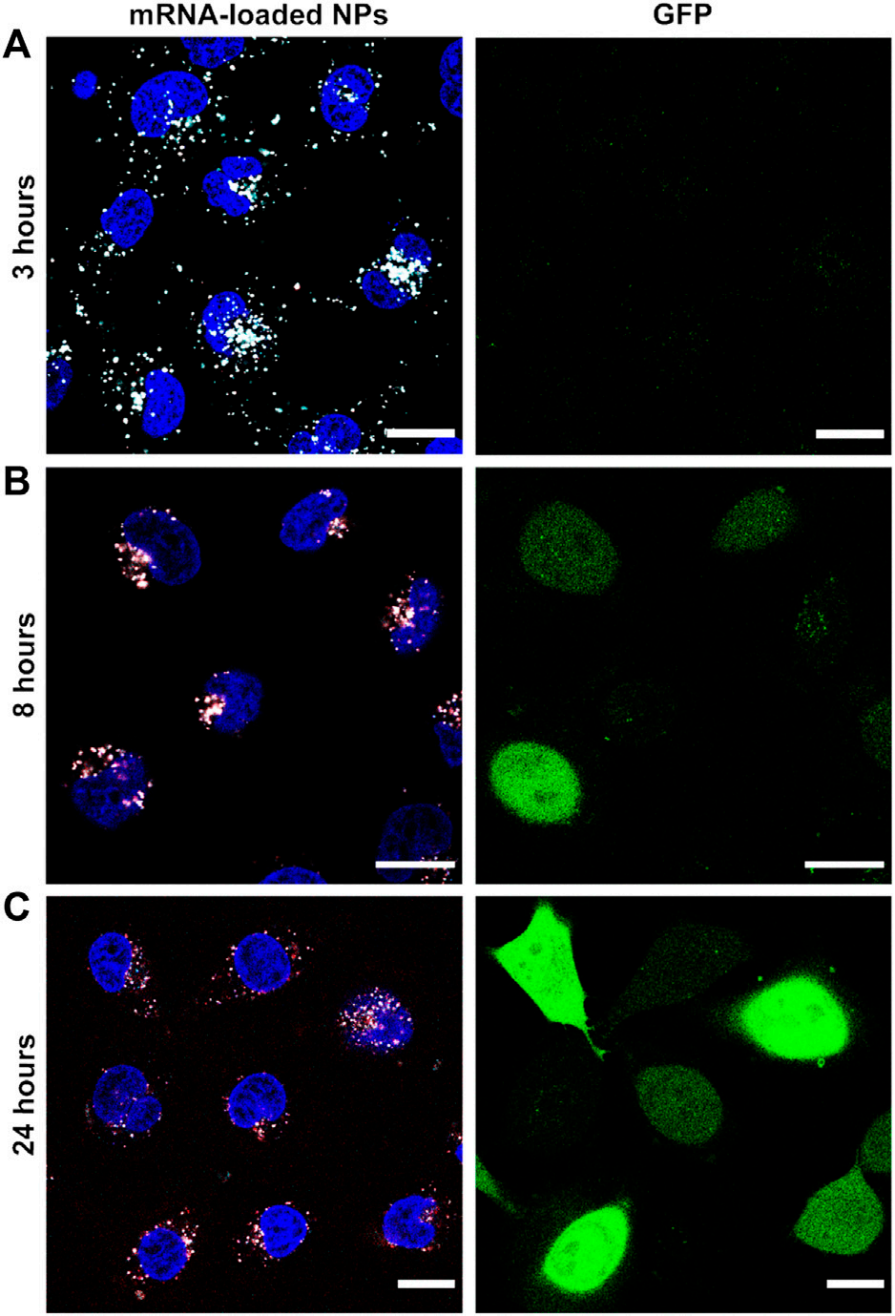
Nanoparticle disassembly and mRNA release in live cells. Fluorescence images of AZDye568-EGFP mRNA release from Cy5-labeled coated ps-PAAQ NPs, after **(A)** 3 h **(B)** 8 h, and **(C)** 24 h of transfection. Loading ratio of 25 w/w ps-PAAQ:mRNA and dosage of 480 ng mRNA per well. The GFP expression is shown in the right column. Nanoparticles are shown in cyan, mRNA in red and colocalization in white color. The contrast of the cyan and red channels was kept constant in all images. Scale bar: 20 µm.

For simultaneous monitoring of nanoparticle disassembly and mRNA release in live cells, Cy5-labeled PEG-coated NPs were co-loaded with a mixture of EGFP mRNA and fluorescent AZDye568-EGFP mRNA. Cells were incubated with these nanoparticles for 3 h, after which the NP-containing medium was replaced with fresh medium, stopping further uptake of NPs. Imaging was performed directly after replacing the medium, as well as after an additional 5 h and 21 h. In the first timepoint (3 h), it was possible to observe bright white spots in the intracellular environment (Figure 6A), which were attributed to ps-PAAQ NPs (cyan) containing AZDye568-EGFP mRNA (red). At longer time intervals (8 and 24 h), the bright spot-like signals arising from the loaded nanoparticles became dimmer (Figures 6B, C) and gradually more disperse over the cytoplasm. This was especially evident after 24 h of transfection (Figure 6C), which suggests that over time mRNA is released from the carrier into the intracellular environment. The GFP expression could not be observed at 3 h after transfection but was detectable after 8 h and was strongest after 24 h, also in accordance with the weaker signal coming from NPs and mRNA.

### 3.5 Effect of modulators of the intracellular reducing environment

**FIGURE 7:**
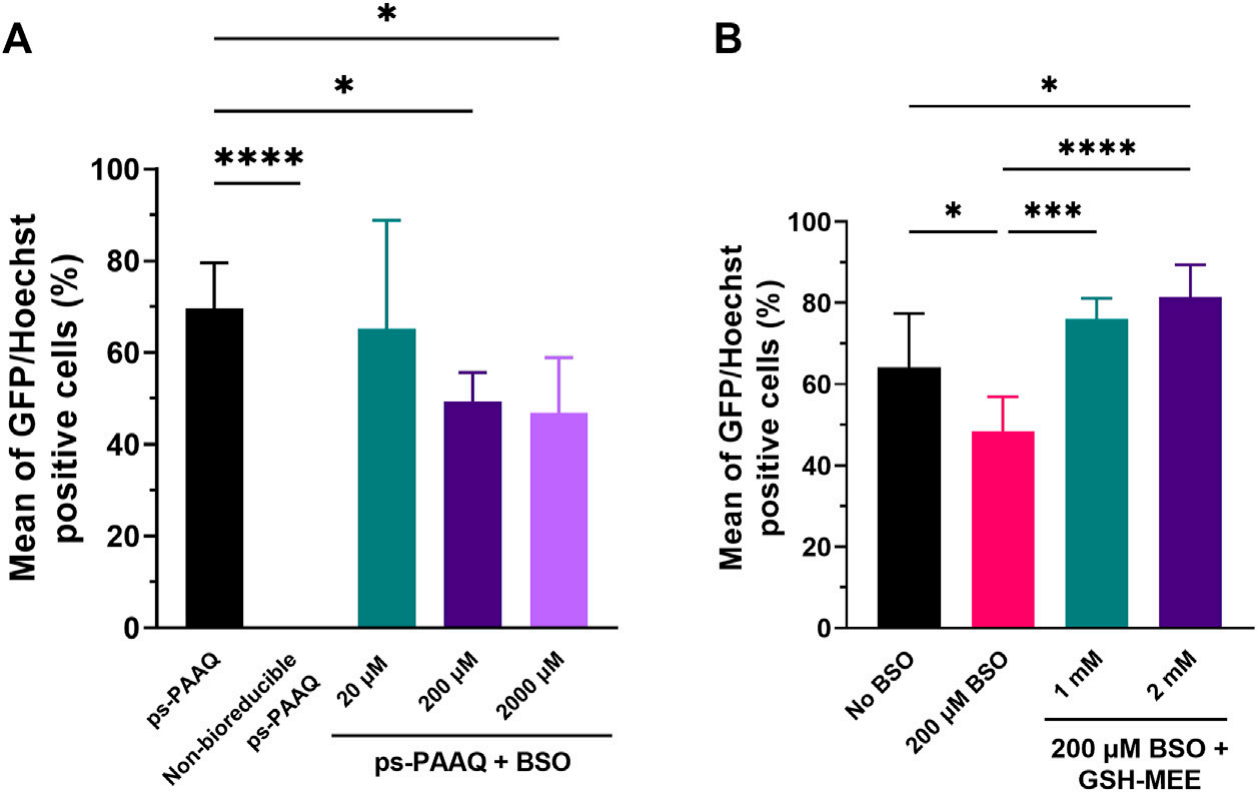
Effect of modulators of the intracellular reducing environment on GFP protein expression. **(A)** Transfection efficiencies after treatment of C28/I2 cells with 20–2000 µM BSO (glutathione synthesis inhibitor) for 24 h prior to transfection with coated ps-PAAQ NPs (8,000–12,000 cells per group, *n* = 3, One-Way ANOVA followed by Dunnett’s test). A negative control group with non-bioreducible ps-PAAQ NPs was also added (without disulfide bonds in the polymer backbone). **(B)** Transfection efficiencies after incubation of BSO-treated chondrocytes with 1–2 mM GSH–MEE for 3 h after medium change (4 h after transfection with coated ps-PAAQ NPs) (8,000–12,000 cells per group, *n* = 3, One-Way ANOVA followed by Tukey’s test). Statistically significant difference is indicated by (*) to *p*-value ≤0.05 (***) to *p*-value ≤0.001, or (****) to *p*-value ≤0.0001.

To assess the effect of GSH depletion on GFP protein expression, we analyzed transfection efficiency after treating C28/I2 cells with an inhibitor of glutathione: buthionine sulfoximine (BSO). This chemical inhibits the activity of glutathione synthetase, an enzyme that catalyzes the last step in the synthesis of glutathione (Drew and Miners, 1984). Chondrocytes were incubated with 20–2000 µM BSO for 24 h prior to transfection, and no significant decrease in gene expression was observed with the lowest dose of 20 µM BSO (Figure 7A). However, higher doses of BSO resulted in a modest but significant decrease in the percentage of GFP-positive cells (49% ± 6% for 200 µM BSO; 47% ± 12% for 2000 µM BSO), compared with the control group not treated with BSO (70% ± 10%). Cell viability was >90% for all treatment groups (data not shown). The importance of the disulfide-mediated release was further confirmed using PEG-coated ps-PAAQ NPs where the disulfide bridges were replaced with an analogous carbon-based methylene linkage that is not susceptible to cleavage. For these carbon-based ps-PAAQ NPs, GFP protein expression was not observed (Figure 7A), even though NP internalization was clearly visible 24 h after transfection (Supplementary Figure S7). These nanoparticles showed comparable size as disulfide-containing coated ps-PAAQ NPs (56.2 ± 2.0 nm; PDI 0.122 ± 0.022). Therefore, these results demonstrate the superiority of bioreducible ps-PAAQ nanoparticles in mRNA transfection in C28/I2 cells.

To prove that the decrease in GFP expression was directly caused by BSO-mediated depletion of GSH, we additionally incubated chondrocytes with glutathione monoethyl ester (GSH-MEE), a cell-permeable derivative of GSH, after treating cells with 200 µM BSO (Figure 7B). The GFP expression levels were recovered after incubating BSO-treated chondrocytes with 1 or 2 mM GSH-MEE, compared to the control group not treated with BSO (64% ± 13%). For the treatment with 2 mM GSH-MEE, the number of GFP-positive cells was significantly higher (81% ± 8%) than the control group without BSO, which implies a greater NP biodegradation and potentially more mRNA release. Cell viability was >90% for all treatment groups (data not shown).

### 3.6 DoE to maximize the transfection efficiency

The optimization of the nanoparticle formulation is crucial to ensure high transfection efficiency while keeping toxicity levels low. In all experiments previously discussed, the same formulation was used, consisting of 25 w/w ps-PAAQ:mRNA ratio and dosage of 480 ng mRNA per well. Although the results were also satisfactory in general for this particular cell line (around 60%–65% of transfection efficiency), we wondered if these outcomes could be further improved by testing different combinations of loading ratios and dosages of nanomaterial in C28/I2 cells. Variables that can impact transfection efficiency were defined as the ps-PAAQ:mRNA ratio and the dosage of mRNA per well. Hereby we applied a D-optimal design to investigate the effect of these parameters on GFP expression by PEG-coated ps-PAAQ NPs, according to the Design of Experiments (DoE) from Table 2. The statistical analysis verified whether the selected variables and their interactions had a significant effect on transfection efficiency in C28/I2 cells. Transfection efficiency >65% was chosen as a critical quality attribute (CQA), considering our results presented so far here. The outcomes were used to establish a design space, an acceptable region within which the quality of the product can be assured (Hakemeyer et al., 2016).

**TABLE 2:** Design of experiments (DoE) to maximize transfection efficiency of coated ps-PAAQ NPs in C28/I2 cells. Transfection efficiencies expressed as the percentage of GFP-positive cells from each combination of ps-PAAQ:mRNA ratio (w/w) and dosage (ng mRNA per well). Triplicates per condition, D-optimal design with 13 runs, including 3 repetitions of the center point N10 (N11-N13) (MODDE 13 software).

Experiment name	ps-PAAQ:mRNA ratio (w/w)	Dosage mRNA/well (ng)	Transfection efficiency (%)
N1	12.5	160	4.48 ± 1.11
N2	50	160	9.58 ± 5.54
N3	25	160	12.90 ± 7.08
N4	12.5	320	35.54 ± 7.91
N5	50	320	42.71 ± 15.43
N6	12.5	640	44.87 ± 5.64
N7	50	640	76.02 ± 11.13
N8	25	640	73.30 ± 14.84
N9	50	480	63.22 ± 8.74
N10	25	480	59.80 ± 7.75
N11	25	480	60.12 ± 7.39
N12	25	480	69.12 ± 7.00
N13	25	480	61.16 ± 12.28

**FIGURE 8:**
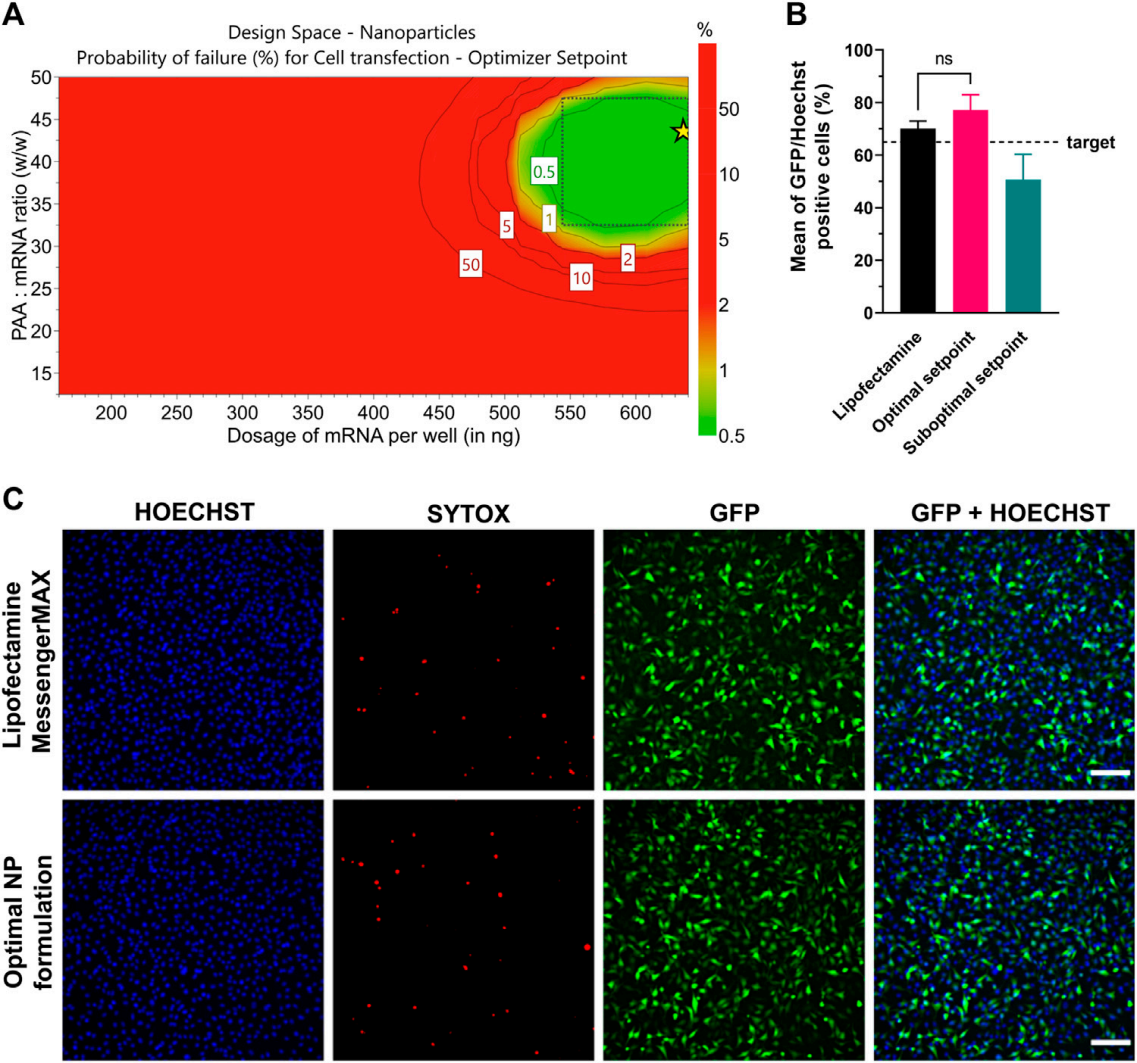
Optimization of transfection efficiency with coated ps-PAAQ NPs in C28/I2 cells. **(A)** D-optimal experimental design for target transfection efficiency >65%, expressed as the error risk for each combination of loading ratios and mRNA dosages. Green area represents error risk ≤5%; red area indicates error risk >5%. Optimal set point indicated by the star symbol **(B)** Optimal formulation (42.5 w/w ps-PAAQ:mRNA ratio and dosage of 640 ng mRNA per well) was validated by three individual experiments. Suboptimal setpoint (25 w/w ps-PAAQ:mRNA ratio and dosage of 400 ng mRNA per well) showed transfection levels below the target of 65% (8,000–12,000 cells per group, *n* = 3, One-Way ANOVA followed by Bonferroni’s test, *p*-value ≤0.05). **(C)** Fluorescence images showing the comparison between the optimal ps-PAAQ NP formulation and Lipofectamine™ MessengerMAX™ as a positive control (following supplier’s protocol). The first column shows nuclear staining with Hoechst; second column represents the dead cells stained by SYTOX™ Orange; third column shows GFP-expressing cells; and the last column displays the merged image. Scale bar: 200 µm.

As a result of the statistical analysis, Figure 8A shows the design space for a target transfection efficiency in an experimental area with low error risk. Each point from the design space surface represents a possible different loading ratio/dosage, having the transfection efficiency >65% as a CQA, with a certain risk level. The risk of getting predictions outside the specifications, expressed as the error risk, was estimated by using Monte Carlo simulations (Figure 8A, red area). As a result, a dose-dependent effect is observed with growing loading ratios and dosages. The optimal setpoint was found at 42.5 w/w ps-PAAQ:mRNA ratio (N/P ratio 34:1) and dosage of 640 ng mRNA per well (Figure 8A, green area). Importantly, no toxicity was observed 24 h after transfection, as cell viability compared to untreated cells was >95% for all tested loading ratios and dosages (data not shown).

The design space was further validated by three independent repetitions of the transfection experiment, comparing the optimal setpoint (42.5 w/w ps-PAAQ:mRNA ratio and dosage of 640 ng mRNA per well) with a randomly chosen suboptimal setpoint (25 w/w ps-PAAQ:mRNA ratio and dosage of 400 ng mRNA per well) (Figure 8B). As a result, all three average measurements from the optimal setpoint were above the 65% target (77% ± 4%; 79% ± 12%; 75% ± 8%), while all three measurements from the suboptimal setpoint were below the 65% target (62% ± 7%; 46% ± 13%; 44% ± 7%). As a positive control, mRNA delivery with the commercially available transfection reagent Lipofectamine™ MessengerMAX™ showed transfection efficiency of 70% ± 3% (Figures 8B, C).

## 4 Discussion

**FIGURE 9:**
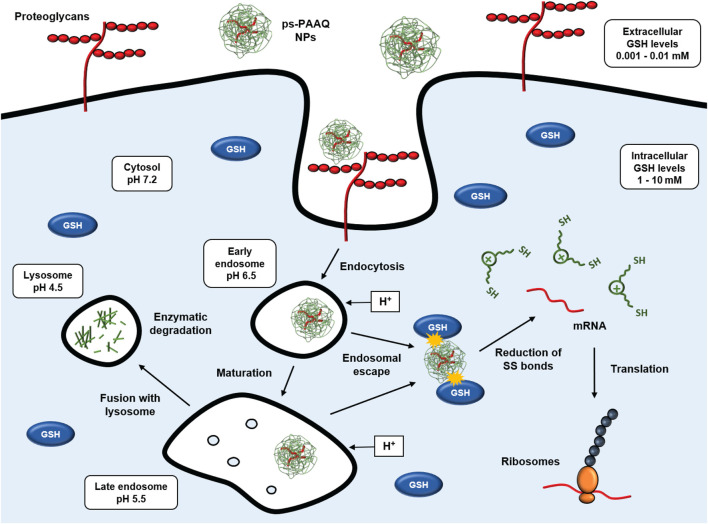
Intracellular trafficking of disulfide-containing ps-PAAQ nanoparticles. Cell attachment is based on electrostatic interaction between positive charges of nanoparticles and negatively charged groups at the cell surface (e.g., heparan sulfate proteoglycans), followed by the endocytosis of the nanocarriers. Once these NPs are internalized into endosomes, they may escape to the cytosol by the so-called “proton-sponge effect”. Finally in the cytosol, the nanoparticle is degraded through reduction of its constituent redox-sensitive disulfide linkages by glutathione (GSH), which enables controlled release of the mRNA and subsequent translation by ribosomes. If endosomal escape does not occur, the late endosome fuses with the lysosome, and the NP is degraded by hydrolytic enzymes.

We studied the intracellular sorting of PEG-coated disulfide-containing ps-PAAQ nanoparticles, and showed evidence of their endosomal escape and controlled biodegradation in live cells. These coated NPs are efficiently taken up by cells, which results in high GFP translation in C28/I2 human chondrocytes. Additionally, the maintenance of the reducing environment in the cytosol was found to be a determinant for NP biodegradation and mRNA translation. Figure 9 shows a theoretical view of the intracellular trafficking of ps-PAAQ NPs investigated in this study and further discussed below.

Cell uptake of the NPs and the influence of PEG-coating on this uptake was first investigated using confocal microscopy. Internalization of the PEG-coated NPs was approximately 50% lower than observed for the uncoated NPs. This finding can be explained by the intrinsic positive charge of uncoated ps-PAAQ NPs, which boosts electrostatic interactions with the negatively charged plasma membrane (e.g., sulfate groups of cell surface heparan sulfate proteoglycans) and facilitates nanoparticle internalization. The addition of the hydrophilic PEG chain coupled to an anionic polymer block (PGA_7.5k_-PEG_5k_) both sterically shields as well as neutralizes the surface charge of the ps-PAAQ NPs and thus reduces the magnitude of this electrostatic interaction, resulting in decreased uptake. These results were further confirmed by FACS as the uptake kinetics of PEG-coated NPs was slower in the first 4 h following transfection. Despite the decreased cell uptake of PEG-coated NPs, surprisingly the resulting GFP expression levels were significantly higher. The higher transfection efficiency shown by PEG-coated ps-PAAQ NPs may be explained by their higher (intracellular) stability and/or different NP degradation/mRNA release profile. Another possible explanation is the difference in particle size when mixed in culture medium. PAA polyplexes are typically prone to aggregation when mixed in culture medium (Vercauteren et al., 2011). In this study we have found that uncoated ps-PAAQ NPs increase in size when suspended in DMEM, possibly by particle aggregation, while in contrast, PEG-coated NPs remained small after incubation (<70 nm) under the same conditions. This may be relevant to their activity, as different particle sizes in combination with different surface charges may change the endocytic pathway of the NPs (He et al., 2010).

Once nanoparticles are internalized into endosomes, they must find their way out to release the cargo into the cytosol and eventually allow protein synthesis. The most accepted mechanism of endosomal escape for cationic polymers is the so-called “proton sponge effect” (Freeman et al., 2013). Previously, PAAs were shown to display buffer capacities superior to PEI under simulated acidic conditions found in endosomes (pH range 5.1–7.4) (Lin et al., 2007), which is proposed to have a considerable influence on the proton sponge effect and therefore on endosomal release. The quinoline moiety (Q) present in the ps-PAAQ composition also contributes to its buffering capacity (Lin et al., 2006). By using calcein as a model for endosomal escape, we observed that there is a dose-dependent effect of the NPs on endosomal leakage. These findings suggest that the higher NP concentrations were needed for an effective build-up of osmotic pressure in endosomes, which in turn increases endosomal rupture and escape of NPs into the cytosol. This outcome is in agreement with the proton sponge effect hypothesis and supports this mechanism in our ps-PAAQ nanoparticulate system. In line with this finding, we were able to quantify endosomal entrapment of NPs over time using LysoTracker™, and showed that it decreased by 50% from 3 to 48 h after transfection.

Payload release from disulfide-bond containing ps-PAAQ NPs into the cytosol depends primarily on the concentration of reduced glutathione (GSH) in the cell (Soundara Manickam and Oupický, 2006). Plasma GSH concentrations are around 1,000 times lower than those typically found inside the cell (1–10 mM) (Forman et al., 2009). The higher GSH concentration in the cytoplasm of eukaryotic cells provides a reducing environment that leads to particle breakdown and payload release. The OA disease progression is characterized by a disturbance in the balanced production of reactive oxygen species (ROS) and antioxidant defenses (e.g., glutathione). Glutathione levels in synovial tissue of MIA-induced rats (monoiodoacetate-induced model of OA) were significantly lower than in healthy animals (Gao et al., 2020), and GSH content in articular cartilage of adult rats significantly declined with age and continuous loading (Zhu et al., 2020). We assessed the impact of lower intracellular GSH levels by depletion of GSH with BSO. Our findings indicate that the transfection efficiency of ps-PAAQ NPs was significantly reduced by BSO although notable GFP expression (>40% positive cells) was still observed. The GFP expression was fully recovered by the addition of the cell-permeable GSH analogue GSH-MEE. The BSO concentrations tested in our experiments were previously shown to be only partly effective in depleting GSH levels in different cell types (Read et al., 2005; Giustarini et al., 2018), since other mechanisms exist to maintain the reduced-to-oxidized (GSH/GSSG) glutathione balance (Suzuki et al., 1997). Furthermore, intracellular polyanion exchange may lead to some payload release without full NP degradation and result in cell transfection (Oupickí et al., 2001). We demonstrated by live cell imaging that ps-PAAQ NPs are highly biodegradable, and that the resulting mRNA release is correlated with a gradual increase in GFP protein synthesis. A similar live tracking was performed with labeled PAMAM-PEG micelles loaded with DNA; however, it showed incomplete polymer dissociation, thus limiting transfection efficiency (Liu et al., 2011). In our study, GFP fluorescence could be observed as early as 8 h after transfection. The mRNA molecules were shown to have a median intracellular half-life of around 10 h in human cells (Yang et al., 2003), what makes the fast and timely translation after release from the carrier an important feature.

The transfection efficiency of the PEG-coated ps-PAAQ NPs was optimized in C28/12 human chondrocytes by DoE. The optimal setpoint (42.5 w/w ps-PAAQ:mRNA ratio and dosage of 640 ng per well) allowed to obtain nearly 80% of transfection efficiency, without observable cell death. The colloidally stabilized PEG-coated ps-PAAQ NPs were effective below the toxic concentrations, which is typically not the case with cationic polymer-derived nanoparticles (Gustafson et al., 2015). Nevertheless, these NPs should be further developed before any therapeutic application in OA. Although some parameters for *in vivo* application may be optimized *in vitro*, such as the polymer-to-mRNA ratio, further optimization of transfection should be performed mimicking the complex composition of the joint. In particular, the dense and highly charged extracellular matrix of the cartilage tissue and the viscous synovial fluid surrounding it are barriers that need to be considered before cell entry. Specificity and efficacy of treatments can then be further improved by targeting to relevant cells in the synovial joint, i.e., chondrocytes if cartilage-specific protein expression is required. Cell targeting may reduce off-target accumulation of nanoparticles that can potentially occur after intra-articular injection in diseased joints. This process is mainly mediated by phagocytosis of the NPs by tissue-resident macrophages, which has been linked to inflammation-induced nanoparticle toxicity (Orr et al., 2011; Gustafson et al., 2015). In this context, the lower uptake observed for PEG-coated ps-PAAQ NPs, compared with uncoated NPs, can additionally be advantageous to improve delivery to chondrocytes in the cartilage, thus preventing the rapid and non-specific uptake by the macrophages that accumulate in the synovial capsule during OA. Furthermore, interactions with components of the synovial fluid can induce changes to the NP surface properties, affecting colloidal stability and influencing the delivery efficacy of the particles (Brown et al., 2019). The neutral surface charge provided by the PEG-coating is expected to lower reactivity with proteins in the synovial fluid.

Although the developed ps-PAAQ NPs proved to be efficient at optimized conditions in a 2D cell culture setting, as mentioned above, further studies are needed to evaluate performance in more complex models simulating or containing cartilage extracellular matrix and ideally synovial fluid. Testing the current formulations in cartilage explant cultures and/or organ-on-a-chip platforms will therefore be a logical next step. Moreover, further evaluation of the NPs in terms of toxicity and finally performance in animal models will be needed. The general biocompatibility and partial optimization of nanoparticle design in C28/I2 chondrocytes paves the way for future experiments in 3D cultures and *ex vivo* or *in vivo* settings.

## 5 Conclusion

In this work the ps-PAAQ nanoparticles showed promising features as a gene delivery vehicle in osteoarthritis research, such as small particle size (about 60 nm), excellent monodispersity and an electroneutral surface charge. Furthermore, we showed high colloidal stability and transfection efficiency in C28/I2 chondrocytes, which was further improved by PEG-coating of the NPs. Importantly, we demonstrated efficient endosomal escape and biodegradation of the PEG-coated NPs. Given the impact of intracellular GSH concentrations on translation, we believe this should be carefully considered when testing bioreducible NPs in the oxidative stressed, GSH-deficient diseased synovial joints. Taken together, our results demonstrate the robustness and biocompatibility of PEG-coated ps-PAAQ NPs in chondrocytes, which holds promise for future studies on optimization of delivery in, and therapeutic mRNA delivery to, cartilage tissue and ultimately treatment of diseased joints.

## Data Availability

The original contributions presented in the study are included in the article/Supplementary Material, further inquiries can be directed to the corresponding author.
