# Supplementary material for: Cell uptake and intracellular trafficking of bioreducible poly(amidoamine) nanoparticles for efficient mRNA translation in chondrocytes
Source: Front Bioeng Biotechnol. 2023 Nov 10;11:1290871. doi: 10.3389/fbioe.2023.1290871 (PMC10668025; doi:10.3389/fbioe.2023.1290871)
Supplement: Supplementary file 1 [file DataSheet1.docx]

Supplementary Material

# 1 Supplementary Tables

Supplementary Table S1. Cy5-labeled NPs characterization by Dynamic Light Scattering (DLS). Results are the combined data of three individual measurements. All nanoparticles were loaded with 60 µg/mL of EGFP mRNA and have a ps-PAAQ:mRNA ratio of 25 w/w.

| **NP Type** | **Z-Average (nm)** | **Polydispersity**  **Index (PDI)** | **Zeta**  **Potential (mV)** |
| --- | --- | --- | --- |
| Coated | 55.0 ± 0.7 | 0.219 ± 0.022 | -3.5 ± 0.8 |
| Coated +  AZDye568 mRNA | 52.3 ± 0.5 | 0.244 ± 0.023 | -3.4 ± 0.7 |
| Uncoated | Low data quality assessment^1^ | | +29.2 ± 2.6 |

^1^ Sample fluorescence – Cy5 dye absorbs at the laser excitation wavelength of the DLS instrument (633 nm).

# 2 Supplementary Figures


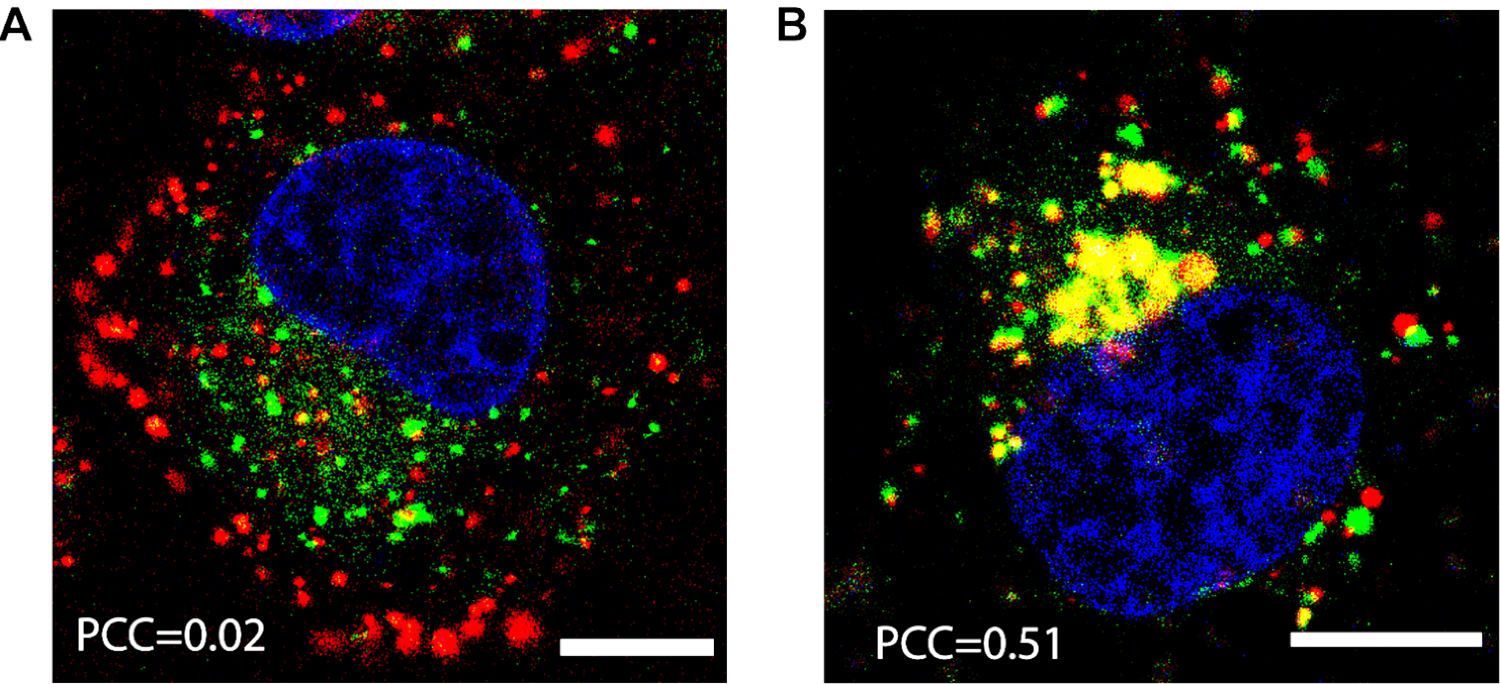


**Supplementary Figure S1.** PCC threshold values. Fluorescence images representing uncorrelated (A) and correlated (B) intensity distribution of the two channels (green for endo/-lysosomes and red for nanoparticles). Scale bar: 10 µm.

PCC values can vary from 0 (no linear correlation between fluorescence intensities of ps-PAAQ NPs and endo-/lysosomes), to 1 (total linear correlation). In order to establish the threshold values of our measurements, we collected both an image where the two intensities were almost excluded, and an image with the maximum colocalization detected. **Supplementary Figure S1A** was recorded 30 minutes after the addition of ps-PAAQ NPs nanoparticles into the medium containing C28/I2 cells, under the confocal microscope. This image was taken as a reference for minimum correlation obtained in the current experiment, corresponding to a PCC value of 0.02. On the other way, **Supplementary Figure S1B** was collected from C28/I2 cells incubated with ps-PAAQ NPs for 3 hours. The PCC value of this image was estimated to be 0.51, representing the maximum correlation reachable in our measurements.


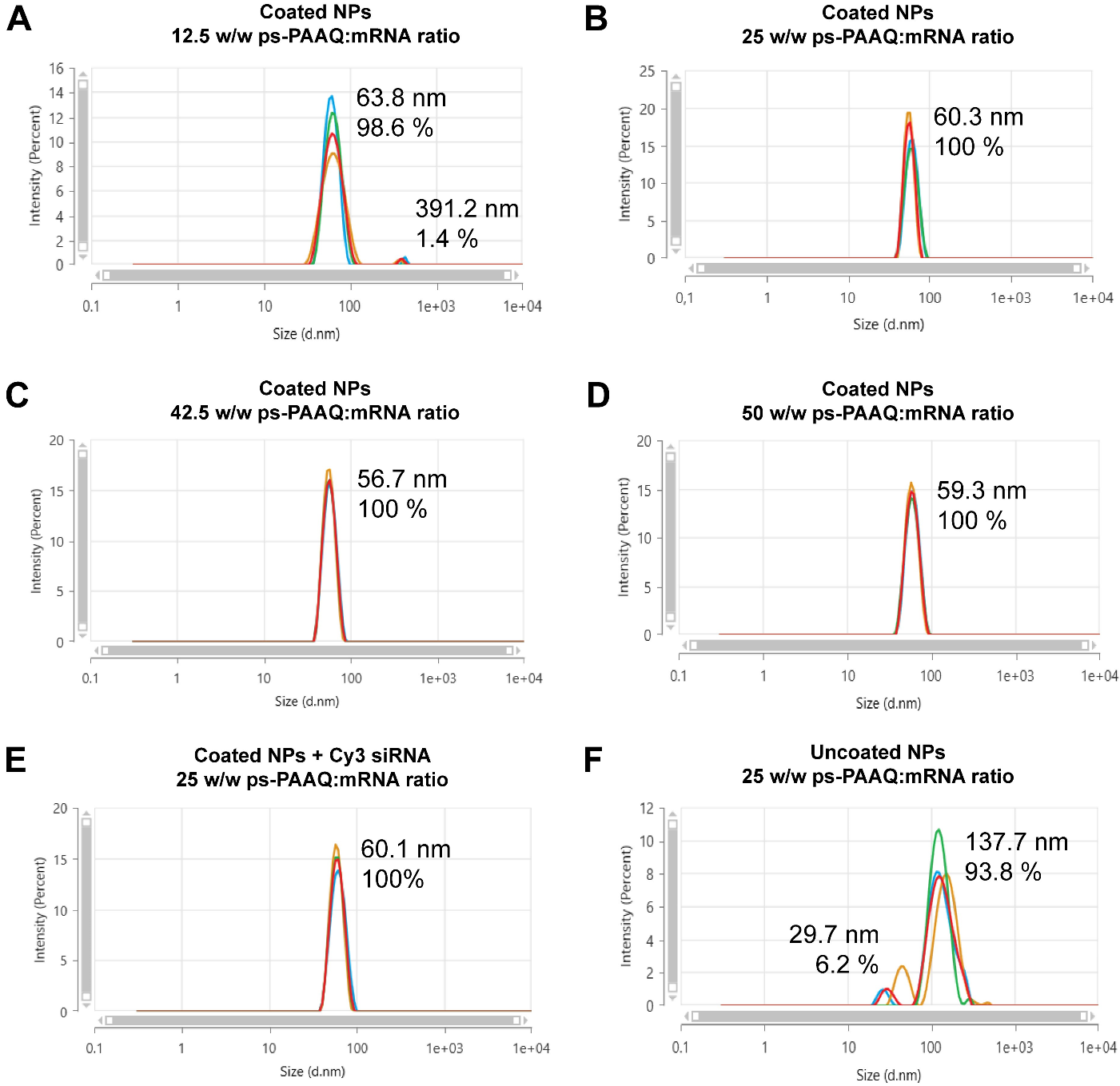


**Supplementary Figure S2.** Particle size distribution of different formulations by Multi-Angle Dynamic Light Scattering (MADLS). Distribution measured by % of intensity and particle diameter (in nanometers), with the indicated % of peak areas. All formulations were loaded with 60 µg/mL of EGFP mRNA. (A-D) PEG-coated ps-PAAQ NPs at different ps-PAAQ:mRNA w/w loading ratios. (E) PEG-coated ps-PAAQ NPs co-loaded with EGFP mRNA and Cy3-labeled siRNA at a 9:1 w/w ratio. (F) Uncoated ps-PAAQ NPs at a single loading ratio. Red curve is the combined data of three angles: 175° (blue), 90° (orange) and 13° (green), with three individual measurements per angle.


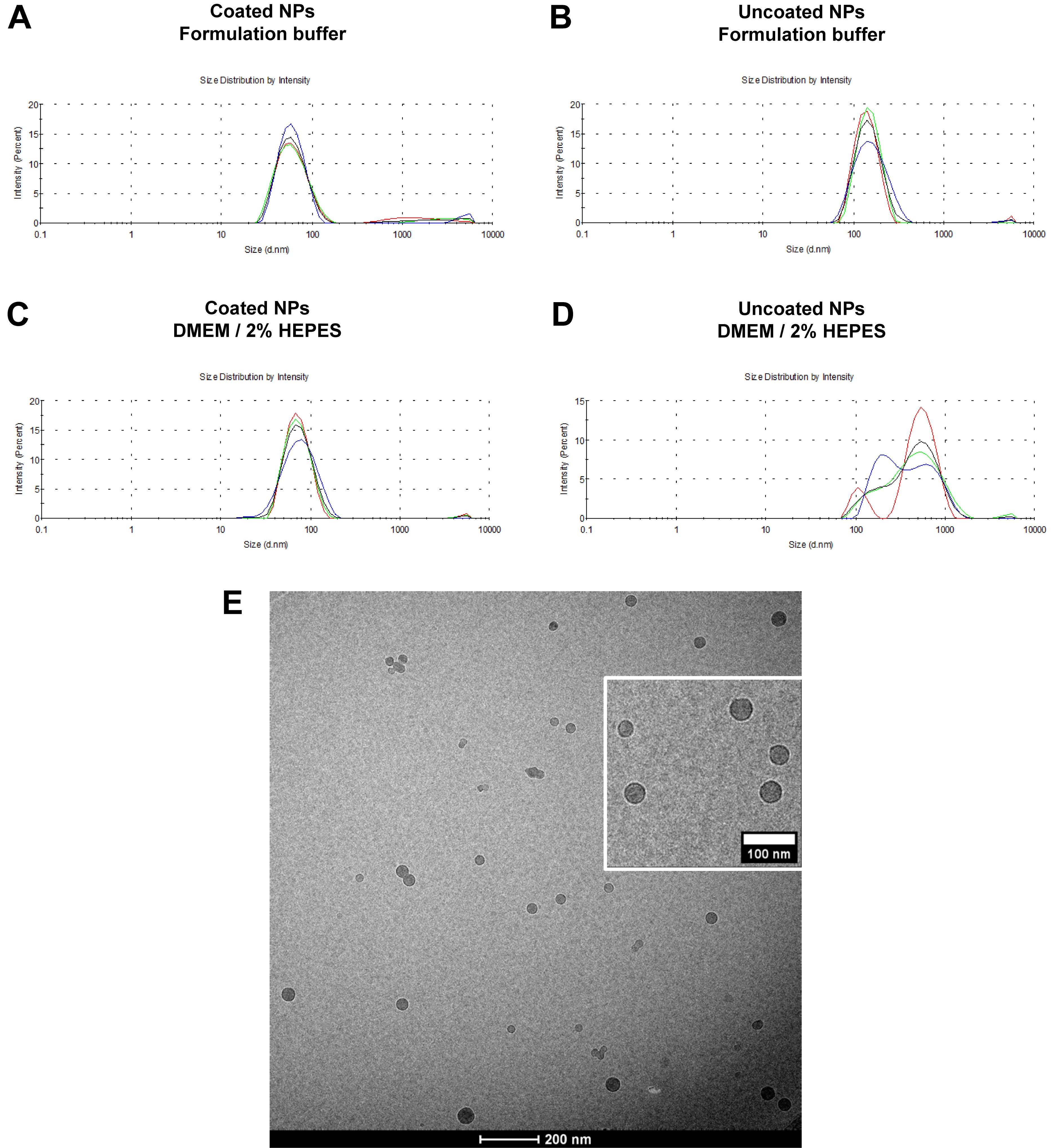


**Supplementary Figure S3.** Particle size distribution by DLS after incubation with formulation buffer or culture medium. Distribution measured by % of intensity and particle diameter (in nanometers). All formulations were loaded with 60 µg/mL of EGFP mRNA. (A) PEG-coated ps-PAAQ NPs incubated with 10 mM Histidine 10% Trehalose buffer. (B) Uncoated ps-PAAQ NPs incubated with 10 mM Histidine 10% Trehalose buffer. (C) PEG-coated ps-PAAQ NPs incubated with DMEM / 2% HEPES. (D) Uncoated ps-PAAQ NPs incubated with DMEM / 2% HEPES. Red curve is the combined data of 3 individual measurements.


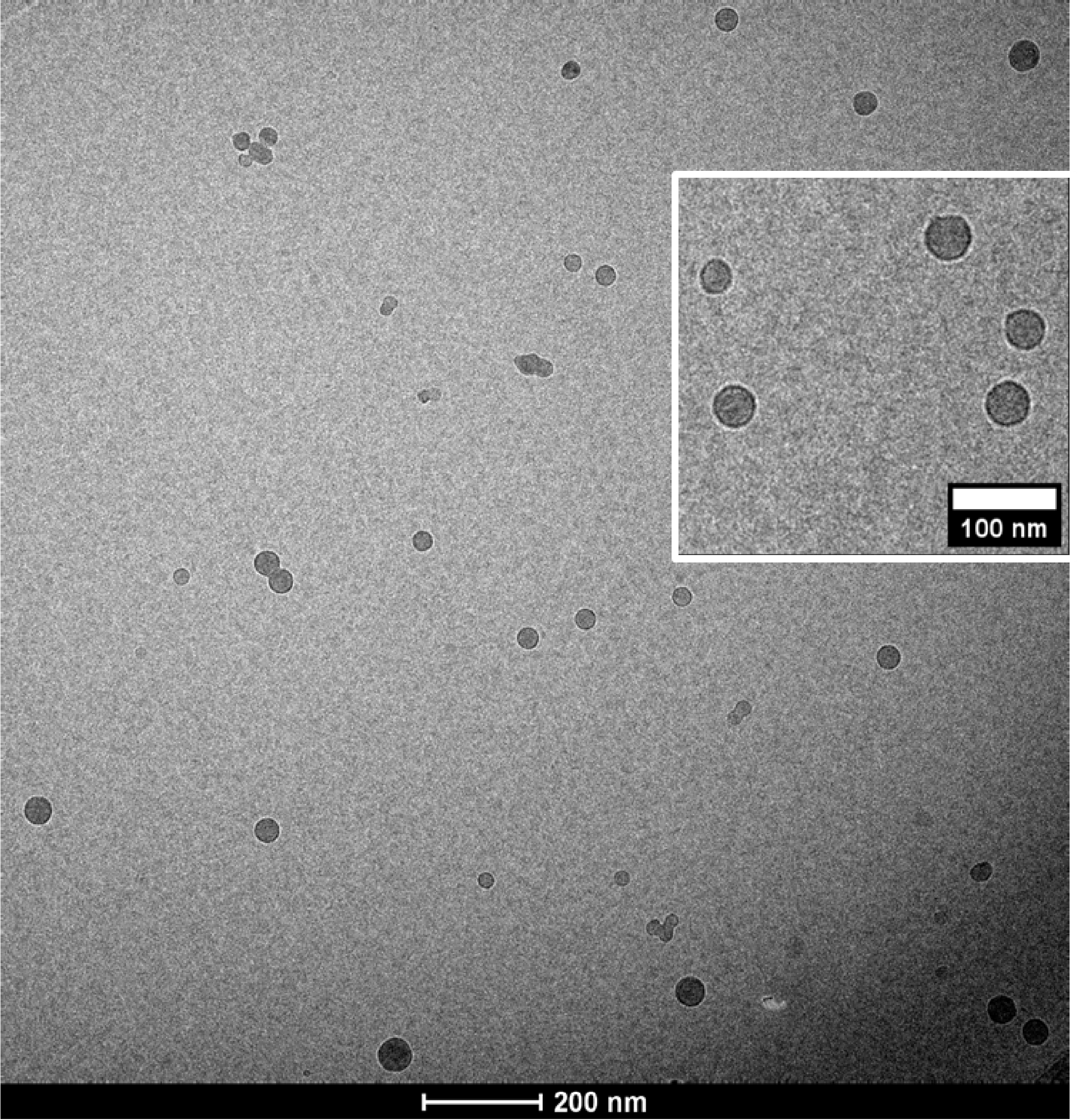


**Supplementary Figure S4.** Cryogenic electron microscopy (Cryo-EM) of the spherical PEG-coated NPs in 10 mM Histidine 10% Trehalose buffer (magnification of 25,000x).


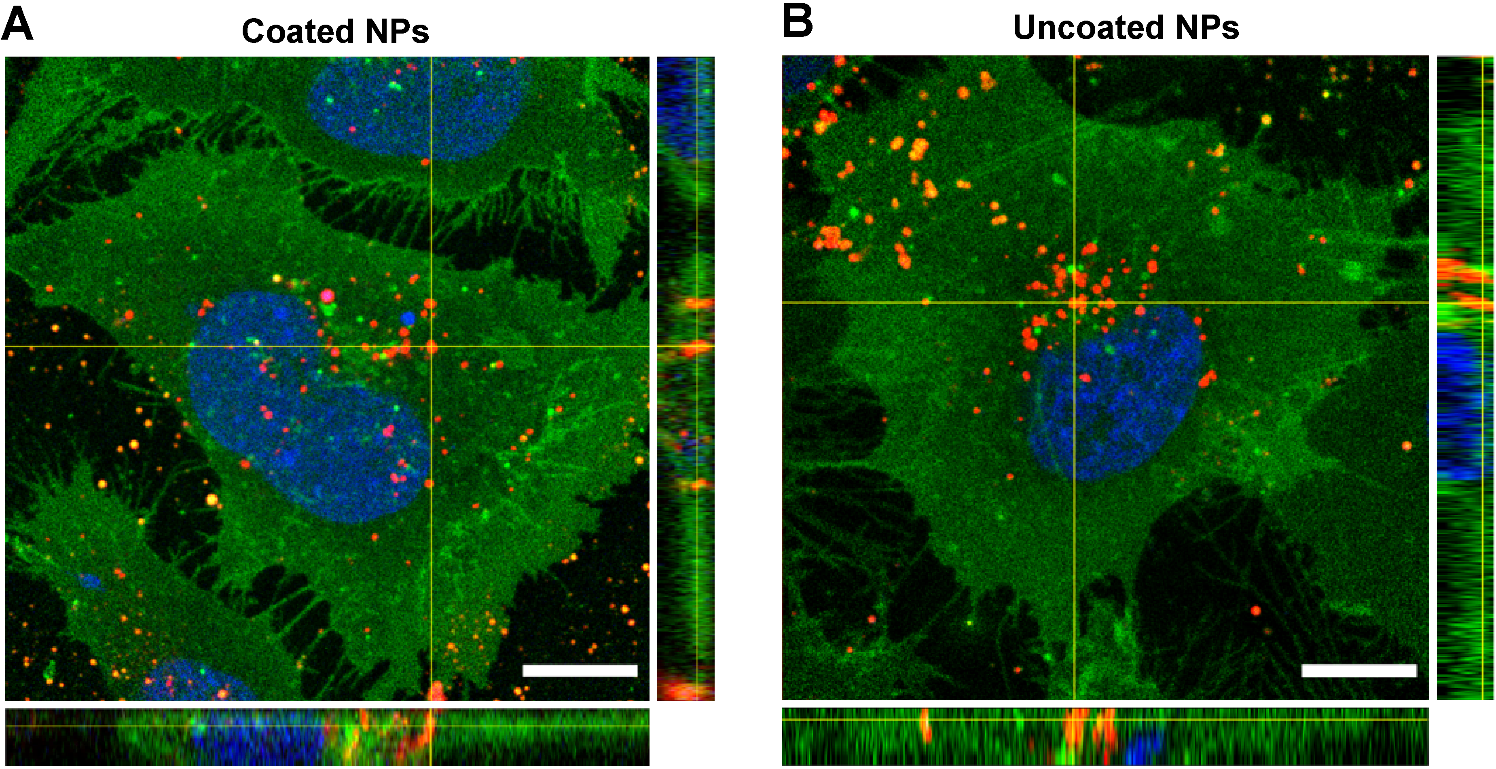


**Supplementary Figure S5.** Orthogonal views of nanoparticle internalization. (A) Uptake of PEG-coated ps-PAAQ NPs. (B) Uptake of uncoated ps-PAAQ NPs. Both nanoparticles were labeled with Cy5 dye (in red). The plasma membrane is stained with CellMask^TM^ Orange and digitally psudeo-colored in green for a higher contrast against the NPs in red. The central panel shows the xy-plane within the cells; the right and bottom panels display the yz and xz projections, respectively. Images acquired 3 hours after transfection. Scale bar: 10 μm.


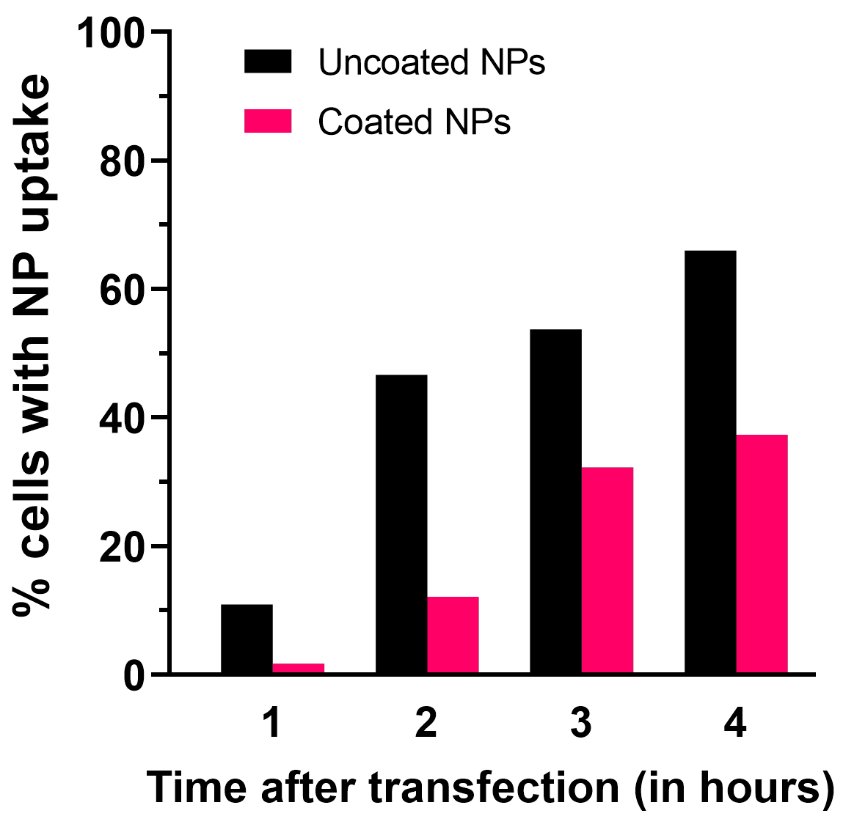


**Supplementary Figure S6.** Uptake kinetics of uncoated versus PEG-coated ps-PAAQ NPs. Nanoparticles were co-loaded with EGFP mRNA and AZDye568-EGFP mRNA at a 9:1 w/w ratio. Transfected C28/I2 cells were fixed 1 to 4 hours after transfection and quantified by FACS using a 561 nm laser line.


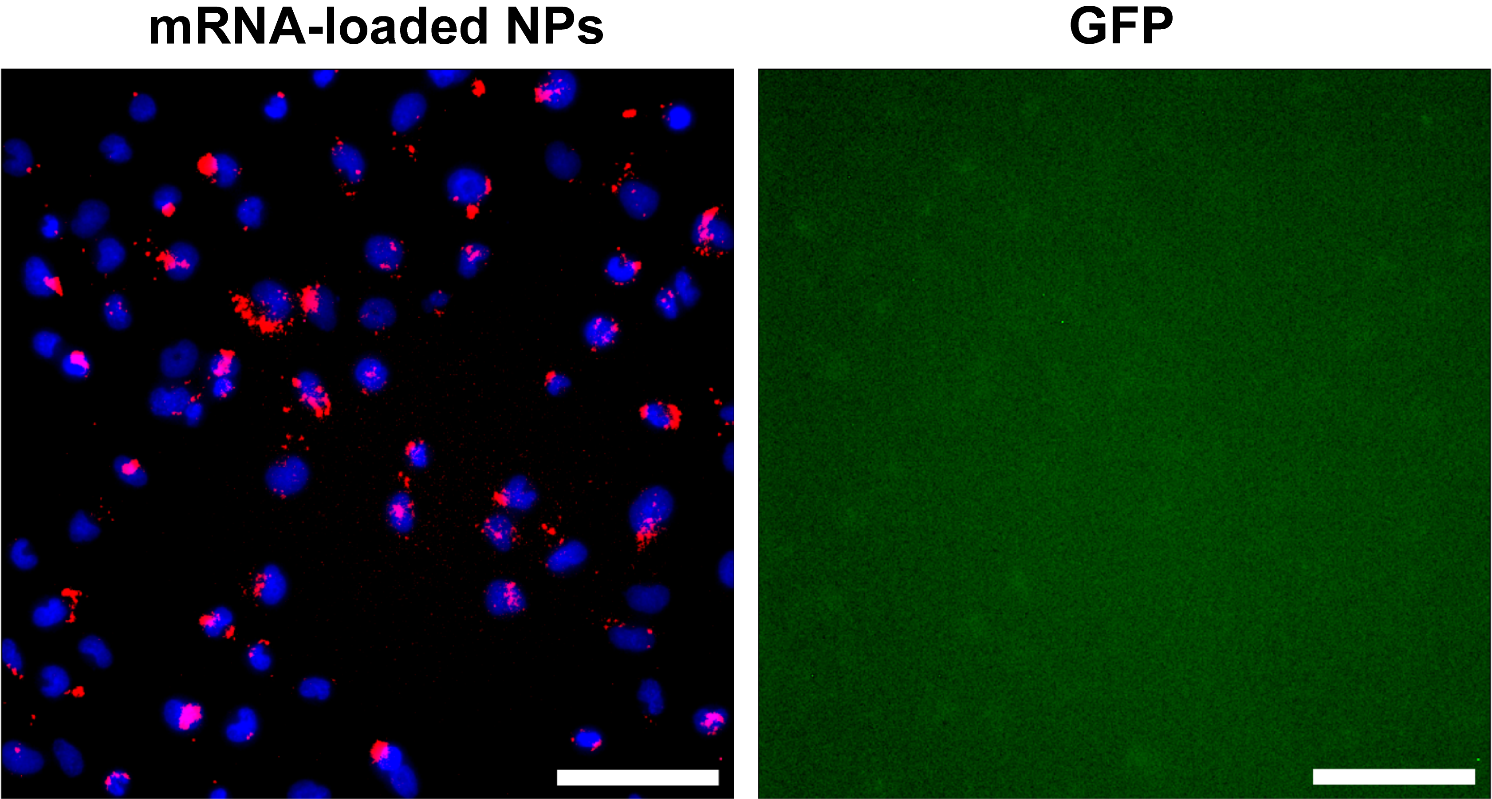


**Supplementary Figure S7.** Internalization of non-bioreducible ps-PAAQ NPs, a carbon-based polymer without disulfide bonds in the backbone. Nanoparticles were co-loaded with EGFP mRNA and AZDye568-EGFP mRNA at a 9:1 w/w ratio. Images acquired 24 hours after transfection of C28/I2 cells. Scale bar: 100 μm.

*Macros*

**1)** Macro function for automated cell counting of the **Hoechst or SYTOX** channels:

 run("Images to Stack", "name=Stack title=[] use"); //convert all images to a stack

//setThreshold(0, 2000); //set threshold values for binary conversion

run("Convert to Mask", "method=Default background=Dark"); //convert stack to binary

run("Watershed", "stack"); //create watershed to separate single cells in clusters

run("Analyze Particles...", "size=100-Infinity pixel show=Outlines clear include summarize stack") //set size limits and launch automated cell counting

**2)** Macro function for automated cell counting of the **GFP** channel:

run("Images to Stack", "name=Stack title=[] use"); //convert all images to a stack

//setThreshold(0, 400); //set threshold values for binary conversion

run("Convert to Mask", "method=Default background=Dark"); //convert stack to binary

run("Remove Outliers...", "radius=2 threshold=50 which=Dark stack"); //set parameters for noise reduction

run("Dilate", "stack"); //dilate areas with weaker GFP signal

run("Watershed", "stack"); //create watershed to separate single cells in clusters

run("Analyze Particles...", "size=100-Infinity pixel show=Outlines clear include summarize stack") //set size limits and launch automated cell counting
